# Promoting prosocial behavior in an unequal world

**DOI:** 10.3389/fpsyg.2022.1021093

**Published:** 2023-02-02

**Authors:** Kelly Kirkland, Jolanda Jetten, Matti Wilks, James Kirby

**Affiliations:** ^1^Melbourne School of Psychological Sciences, University of Melbourne, Melbourne, VIC, Australia; ^2^School of Psychology, University of Queensland, Brisbane, QLD, Australia; ^3^Department of Psychology, School of Philosophy, University of Edinburgh, Edinburgh, United Kingdom

**Keywords:** compassion, superordinate behaviour, norms, cooperation, competition, inequality

## Abstract

Amid a global pandemic and the climate crisis, there is an increasing need to understand how to promote largescale, coordinated action between different groups. Yet certain factors such as inequality can hinder cooperation. We aimed to establish how to orient groups toward a superordinate goal when they have unequal resources. Participants were divided into two ‘countries’ and asked to assemble LEGO bricks into food (by building pieces in a certain order) to prevent starvation among ‘the people’. One ‘country’ had few LEGO bricks whereas the other had an abundance, and the only way to maximize food creation was for the groups to work together. We assessed the efficacy of three diverse interventions on superordinate behavior and attitudes: compassion meditation training (Study 1), lower inequality (Study 2), and the introduction of a pro-sharing group norm by a confederate (Study 3). Compassion meditation training and altering the degree of inequality between groups did not have a clear effect on collaborative action. Only the introduction of a pro-sharing group norm enhanced sharing behavior, made participants feel more cooperative and reduced fears of being compassionate toward others. Our findings speak to the importance of leadership in promoting coordinated action to address challenges that face the superordinate group.

## Introduction

In the 21st century, we are facing large scale collective action problems that may have catastrophic consequences – from vaccine hoarding in a global pandemic to the ongoing refugee crises around the globe (Casey, 2021; Rouw et al., 2021). It is clear that, in order to solve these crises, we need compassionate and coordinated action from the global community – that is, we need to act as one. However, reality tells us that we do not always adopt superordinate goals such as these, particularly when our interests conflict with those who are different from us. Indeed, as classic social psychological research has shown, strong ‘us’ versus ‘them’ dynamics undermine the potential for coordinated action to achieve superordinate goals (Sherif, 1958). Such intergroup divisions are all the more difficult to bridge when there are high levels of inequality between groups whereby one group has more resources than another (Sánchez-Rodríguez et al., 2018). In the current work, we study two unequally resourced groups that have the option to work together to achieve a superordinate goal. Across three studies, we explore whether diverse interventions (individual, structural, and normative) can overcome barriers to compassionate and cooperative action across group boundaries.

### Superordinate goals help to overcome the intergroup divide

Muzafer Sherif was one of the first to discuss the importance of superordinate goals – goals where two or more groups need to cooperate to achieve a particular outcome (Sherif, 1958; Haslam, 2018). In the Robbers Cave experiment, Sherif coordinated a summer camp with several young boys where they were separated into two groups. Unbeknownst to the boys, the summer camp was a disguise for a larger goal – to explore how individuals compete and cooperate when they are members of different groups. The two groups quickly fell into conflict, and cooperation could only be achieved when the experimenters introduced a superordinate goal. The findings show that when people are divided into groups, intergroup conflict can arise, and individuals often pursue the goal of their ingroup at the expense of their outgroup counterparts (Gaertner et al., 2000).

Since these original studies, research has reinforced that an intergroup divide can be bridged when groups adopt superordinate goals (Sherif, 1958; Gaertner et al., 2000; DiBenigno, 2018; Martinez-Ebers et al., 2021). However, there are many socio-structural factors that prevent group members from embracing such superordinate goals. One obstacle might be the magnitude of the resource gap between the groups. If this is perceived to be too large, group members at opposite ends of the wealth spectrum may see the groups as too different and this might affect coordinated action (Jetten and Peters, 2019). Group resource inequality not only enhances the perceived difference *between* groups (e.g., the rich and the poor become more distinct categories, it also increases perceptions of similarities *within* groups (e.g., those within a poor category are perceived to be more similar, Jetten et al., 2017). Indeed, resource inequality enhances ‘us’ versus ‘them’ divisions (Sánchez-Rodríguez et al., 2018), and there is evidence that inequality, therefore, leads to less compassionate behavior toward others (Côté et al., 2015; Sands, 2017; Kirkland et al., 2019, 2021; Tanjitpiyanond et al., 2022).

Failing to overcome the ingroup-outgroup divide can have negative consequences when groups need to cooperate to achieve superordinate goals. We are interested in investigating potential interventions to reduce intergroup competition in this context—with the ultimate aim of increasing compassionate action in line with a superordinate goal. We focus on three possible interventions. The first targets individualistic solutions and we test the power of compassion training aimed at making individual group members feel and act more compassionately. Our reasoning builds on past research that has shown that compassion training (i.e., engaging in compassion meditation exercises), can foster compassionate action (Leiberg et al., 2011; Condon et al., 2013; Trautwein et al., 2020).

Second, interventions that change the structural context may offer an effective method to elicit compassion by altering or shifting group dynamics. We reason that if, relative to low economic inequality, high economic inequality exacerbates the ingroup-outgroup divide (Sánchez-Rodríguez et al., 2018) and erodes prosocial behavior (Côté et al., 2015; Sands, 2017; Kirkland et al., 2020), reducing the degree of inequality may foster greater adoption of superordinate goals.

Third, another potentially effective intervention focuses on informal leaders altering group norms that promote acting for the common good and breaking down ‘us’ versus ‘them’ barriers. Leaders that focus on achieving desired superordinate outcomes might be particularly influential when there are no established group norms and when people therefore look to others for appropriate actions on how to behave (Jetten et al., 1996; Smith and Louis, 2008, 2009; Orlando, 2020). Past research has demonstrated that group norms can have a significant impact on the behavior of other group members (Reicher et al., 2006; Tarrant et al., 2009; Nook et al., 2016; Lay et al., 2020), and may influence the adoption of superordinate goals.

### The present research

If we are to understand how to foster compassionate action in the face of large-scale problems, we need to understand what individual, structural and normative factors may shift group behavior. The current study aimed to gain a better understanding of effective ways of orienting individuals toward superordinate, compassionate behavior when they are embedded in an unequal intergroup context. In particular, we assessed the efficacy of compassion meditation training (Study 1), lower levels of structural inequality (Study 2), and the introduction of a pro-sharing group norm by an informal leader (Study 3). If we are to tackle some of the pressing issues of the 21^st^ century such as climate change and future pandemics, we need to establish which interventions are most likely to lead to collaborative action that gives priority to superordinate goals.

## Study 1

Several scientific studies have demonstrated that compassion meditation practices can promote prosocial behavior and compassionate responding (Leiberg et al., 2011; Condon et al., 2013; Weng et al., 2015; Luberto et al., 2018). To date, most of these have demonstrated an effect of long-term compassion meditation practice (eight-to-nine weeks), but there is less research about the effect of short-term compassion meditation on behavior. Brief interventions, such as 10-min meditations (Kirby and Baldwin, 2018), as well as self-compassion re-framing have led to changes in self-report levels of motivation and feelings (Chwyl et al., 2021), but the researchers did not assess how behavior might be impacted. Past work has also shown that cueing individuals to the needs of others results in more prosocial behavior in dictator games (Brañas-Garza, 2007). In each of these studies however, an individual is typically asked to make a compassionate decision individually, yet little is known about whether compassion meditation can affect decisions made by groups, and specifically, group contexts where there is an unequal distribution of resources. This is important to understand as many compassionate acts in the real world need to be made by groups rather than individuals, and also occur in a broader ecological context.

Here we aimed to assess the effect of a brief compassion meditation on the adoption of a superordinate goal when groups are unequal. To achieve this, we exposed participants to one of two 10-min meditation exercises: compassion meditation or a focussed imagery meditation. Participants were then divided into groups and asked to complete a food assembly task to make food for starving people. As exposure to compassion meditations appear to enhance compassion toward others (Leiberg et al., 2011; Trautwein et al., 2020), we hypothesised that all participants would behave more compassionately by working collaboratively after being exposed to a compassion meditation compared to a focussed imagery meditation.

### Method

Ethical clearance of the study was obtained in line with the ethical review processes of the Human Research Ethics Committee (protocol number: 2018002500).

#### Transparency and openness

This study, including the hypotheses and analytical approach, were preregistered on the Open Science Framework (OSF). We note in the method and results sections below which measures were confirmatory and exploratory. All data, materials (where feasible) and R script has also been made available on OSF. See the following link for these resources: https://osf.io/fjp6b/?view_only=b51cca6755654a76b8d1c8d77a3cfa53.^1^ For each study, we report all data exclusions (if any), all manipulations and how the sample size was determined.

#### Design summary

Our study design was inspired by an activity used with high school students in a non-scientific setting (Schairer, 2018). *Compassion It*—an organization that aims to invoke more compassionate action in the world—ran compassion workshops with students in grade 10 in San Diego, USA. At the conclusion of these workshops, the students were assigned to groups that represented high (e.g., USA) and low (e.g., Dominican Republic) income countries. All countries were tasked with the same goal, to produce as much ‘food as they could’ using LEGO bricks in a specified time. The countries either had an abundance (high income) or not enough (low income) LEGO bricks. Critically, how they should go about achieving this goal was ambiguous; the teenagers were not told whether they should compete or cooperate with the other nations. The students were free to move around the room during the exercise and observe the other countries and their resources. During the exercise, none of the high-income countries spontaneously shared any LEGO bricks with the low-income countries even when those low-income countries asked for help. This suggests that inequality may be a suppressor of behavior in line with a superordinate goal. However, even though these findings are noteworthy, the activity was designed as a learning opportunity rather than a scientifically valid study and these findings should be interpreted with caution given the lack of experimental control.

In the current study, we transformed the activity by *Compassion It* into a rigorous and highly controlled experimental design (Schairer, 2018). Participants were randomly assigned to groups that represented either a high or a low resource country and each country was presented with LEGO bricks, whereby the high resource group had an abundance of LEGO bricks and the low resource group had very little. Participants were asked to assemble LEGO bricks into food items to ensure that “no one will starve.” Critically, the means by which participants should achieve this goal was ambiguous; we did not tell the groups to compete or cooperate.

The compassionate, superordinate goal was to maximize the food creation to ensure no one will starve, and any behavior that contributed to this goal (e.g., working together) was considered an indication of compassion. To achieve this, we coded for three forms of compassionate behavior: (1) initiating sharing of items, resulting in a transfer of LEGO bricks from the high resource group to the low resource group, (2) the amount of food pieces made, and (3) the efficiency (i.e., speed) of food making. We further included several self-report measures that broadly measured competitive and cooperative attitudes toward the other group, group cohesion and fears of showing and receiving compassion.

#### Participants and design

The sample was comprised of 283 participants (178 female, 103 male, 1 gender-diverse, 1 no response) and were 21.36 years-old on average (*SD* = 4.28). Based on a sample size calculation on Pangea, we required a minimum sample size of 152 to detect a medium effect size with 80% probability. This effect size was deemed appropriate based on findings regarding competitive sentiments under situations of high inequality (Sánchez-Rodríguez et al., 2018). We aimed to achieve this sample size at a minimum and collected larger numbers until the participant pool was exhausted. The data was not analyzed until data collection ceased. Participants were recruited from either a first-year pool of psychology students from a large urban university (in exchange for course credit) or from a paid-pool and were reimbursed $10 per half an hour of participation. Participants reported several demographic variables including their age, gender, level of education, ethnicity, total pre-tax income and subjective SES. Subjective SES refers to where one feels they fit into society relative to others in terms of job prestige, education, and income on a 10-rung ladder (where 1 = bottom of society and 10 = top of society). On average, participants reported having middle class backgrounds (*M* = 5.09, *SD* = 1.81). See Supplementary materials 2 for the full demographic description of the sample used.

The current study employed a 2 (condition: compassion meditation, focussed imagery meditation) by 2 (resource group: high, low) between-subjects design, and participants were randomly assigned to a condition and resource group. We were interested in the effect of these independent variables on outcomes described in greater detail below.

#### Procedure

Before the study began, the table and chairs were arranged in a way to clearly separate two groups: the high resource group and the low resource group. Each session contained between 4 and 12 participants. In the event of odd numbers, the extra participant was assigned to the high resource group. The table held two transparent containers with LEGO bricks, with one container assigned to each resource group. Images of these containers can be provided to readers upon request from the corresponding author. The high resource group container held 500 colored LEGO bricks (red, yellow, green and blue) and 100 non-colored bricks (black, white, grey, beige and brown). In contrast, the low resource group container contained only 100 colored bricks and 500 non-colored LEGO bricks. Importantly, the valued resource in this context was colored LEGO bricks, whereas non-colored bricks held no value.

As participants entered the room, they were randomly assigned to sit at the high or low resource group side of the table (see Supplementary materials 3 for the randomization procedure). After consent was obtained, participants were asked to listen to a 10-min meditation audio track which was played aloud to the entire group. Each session was randomly assigned to engage with a compassion meditation track or a focussed imagery track. The compassion track began with basic meditation instructions, before telling participants about the definition of compassion, asked them to contemplate the definition and imagine engaging in compassionate behavior. Our focussed imagery meditation condition served as an ideal control task, as it contained basic meditative practices (e.g., focussing on breathing and one’s body in space) but did not contain any information about compassion. In line with past approaches (Hutcherson et al., 2008; Kirby and Baldwin, 2018), this allowed us to isolate the effect of reflecting on being compassionate from the practice of general mindfulness. See Supplementary materials 4 for full scripts of each meditation.

The experimenter then told participants they were separated into two ‘countries’: Nasherland and Lindithia (see Supplementary materials 5 for the full script). We chose fictitious countries as real countries may prime stereotypes in participants’ minds about behavior in that specific culture and the use of fictitious nations has been successful in past experiments (Jetten et al., 2015; Sprong et al., 2019). Participants read a basic description of their country which contained demographic information such as the local delicacy, the population and the climate (see Supplementary materials 6 for the country descriptions). The experimenter then told participants the aim was to create as much food as possible within a 5-min period to prevent starvation. Participants were told one piece of food could be created by assembling LEGO bricks in the following order (from bottom to top): blue, green, yellow, red. Participants were then shown an image of a correctly assembled food item. This image can be provided to readers upon request from the corresponding author. These instructions were purposefully ambiguous; we did not tell the groups to cooperate or compete as we were interested in how they would interpret the ambiguous situation. As such, if participants asked if they could share bricks, they were told “The aim is to make as much food as possible so no one will starve.” We pilot tested the LEGO brick distribution to ensure the high resource group could not finish assembling their LEGO bricks in the time given whereas the low resource group would always finish assembling their pieces with excess time left. Implicitly, it was clear that the only way to maximize food creation was for the groups to work together.

The groups then had 5 min. to assemble food, and the participants were then asked to complete a questionnaire at the conclusion of the task (see Supplementary materials 7 for full questionnaire given to participants).^2^ Participants were then debriefed and thanked for their participation.

#### Measures

##### Compassionate behavior

We defined compassionate behavior as actions that would contribute to the superordinate goal of creating food for the starving people more broadly. Here, any action that results in maximizing food creation preventing starvation (as this would reduce suffering) was counted as compassionate behavior. First, we assessed whether individuals initiated sharing (yes or no) as well as the amount of LEGO bricks that were transferred from the high to low resource group per individual. Importantly, sharing could be initiated by the high or low resource group. Table 1 demonstrates the kinds of behavior that were counted as initiating sharing per resource group, and whether that instance of sharing was initiated by the high or low resource group. This coding system meant that both the high and low resource group could engage in the sharing of LEGO bricks between the groups. In addition, we assessed the number of correctly assembled food pieces made per individual. Finally, we assessed the food making efficiency (number of pieces assembled per minute) of each resource group.^3^

**Table 1:** Behaviors that were or were not considered as initiating sharing, by resource group for all studies.

	High resource group	Low resource group
*Sharing*	Shares LEGO brick/s spontaneously	Requests high resource group to share LEGO brick/s, and high resource group shares
	Pools LEGO bricks with low resource group	Takes LEGO brick/s from high resource group and high resource group allows it
		Pools LEGO bricks with high resource group
*No sharing*	Discuss sharing within group	No request and no taking of LEGO brick/s from other group
	Vague response to low resource group request, and no clear giving	
	No response to low resource group request	
	No offer to low resource	

Participants could be classified as engaging in more than one behaviour.

##### Fears of compassion

The questionnaire contained a fears of compassion scale (Gilbert et al., 2011), as past work has found people can be fearful of being compassionate to others because it could result in resource loss (Gilbert et al., 2011) and be fearful of receiving compassion from others due to obligations to return the help (Cameron et al., 2019). We included these measures as exploratory additions to the study, as fears of giving and receiving compassion may be a significant barrier to coordinated action. Participants were asked 10-items that reflected fears of *giving* compassion (e.g., “People will take advantage of me if they see me as too compassionate”) and 13 items that gauged fears of *receiving* compassion (e.g., “I worry that people are only kind and compassionate if they want something from me”). Responses per item were scored from 0 (*do not agree at all*) to 4 (*completely agree*), and the responses were added together for each participant to achieve a total score. For fears of giving compassion, the total score could range from 0 (*least fear*) to 40 (*greatest fear*), and for fears of receiving compassion, the total score could range from 0 (*least fear*) to 52 (*greatest fear*). The fears of giving and fears of receiving compassion scales both yielded acceptable reliability (α = 0.84 and α = 0.87, respectively).

##### Group dynamics and cohesion

In the current study, the means (i.e., compete or cooperate) by which the groups should achieve the goal (i.e., create as much food so no one starves) was purposefully ambiguous. To assess the participants’ interpretation of these ambiguous instructions, we included three exploratory questions to ascertain whether they interpreted the task as competitive or cooperative. Participants were asked “To what extent did you feel this task was a competition between the two countries?,” “To what extent did you feel this task was a cooperative task between the two countries?” and “To what extent did you feel the context was one of “US” (my group) versus “THEM” (the other group).” Responses were scored on a scale from 1 (*not at all agree*) to 10 (*strongly agree*). We further asked two exploratory questions to gauge how participants felt about the cohesiveness of their group, as strong ingroup unity may act as a suppressor of coordinated action. Specifically, participants were provided with the following statements: “I felt a sense of unity within my group” and “I felt that people in my group seemed to be on the same wavelength.” Responses were scored on a scale from 1 (*not at all agree*) to 10 (*strongly agree*), and an average score of these two items was created (*α* = 0.86).

##### Attention checks

Finally, participants were asked several questions probing their attention to the inequality as well as a manipulation check to assess feelings of compassion. First, inequality salience was measured with the following question: “During the activity, to what extent did you notice the groups were unequal?.” This question was scored on a scale from 1 (*not at all aware*) to 10 (*extremely aware*). Second, we included a measure to ensure the high resource group felt like they had a greater capacity to complete the task compared to the low resource group: “My group had enough LEGO bricks to complete the task.” Responses were scored from 1 (*not at all agree*) to 10 (*strongly agree*). Participants were also asked “To what extent did listening to the audio track make you feel more compassionate?,” and responses were recorded on a scale from 1 (*not at all agree*) to 10 (*strongly agree*).

#### Analytical approach

In our design, individual behavior was potentially impacted by the behavior of their group members. For example, if one group member decided to share, this may have influenced other group members to share as well. To adjust for this non-independence of data, all individual level measures were analyzed in Linear Mixed Models with ‘group’ (i.e., the specific resource group one was a part of) as the random intercept.

### Results

See Supplementary materials 8 for the full results for each analysis, including mean differences between conditions and resource groups. An independent samples *t*-test showed that there was no significant difference in the sizes of groups randomly allocated to the compassion meditation and focussed imagery conditions, *t*(280.99) = 1.32, *p* = 0.187. This variable was thus not considered further. The conditions and resource groups also did not differ in terms of age and gender. See Supplementary materials 9 for the means and standard deviations per condition, per resource group for each of the dependent variables.

#### Attention checks

Overall, participants were highly cognizant of the unequal resources between the two groups (*M* = 6.66, *SD* = 3.19). An LMM was conducted on the effect of resource group and condition on the extent to which the participants noticed the inequality. There were no differences between resource groups, *F*(1, 56.89) = 0.50, *p* = 0.482, or conditions, *F*(1, 56.89) = 2.28, *p* = 0.137, in the extent to which participants noticed the inequality. Likewise, there was no significant interaction between resource conditions and compassion manipulation conditions, *F*(1, 56.89) = 3.45, *p* = 0.069.

We further assessed the extent to which participants felt they had enough LEGO bricks to complete the task. An ANOVA^4^ revealed a significant effect of resource group, *F*(1, 278) = 313.62, *p* < 0.001, where the high resource group (*M* = 8.71, *SD* = 2.12) indicated more strongly than the low resource group (*M* = 3.62, *SD* = 2.68) that they had enough LEGO bricks to complete the task. However, there was no significant effect of condition, *F*(1, 278) = 0.03, *p* = 0.852, and no condition by resource group interaction, *F*(1, 278) = 0.04, *p* = 0.846.

Finally, we assessed whether participants felt more compassionate after listening to the compassion compared to the focussed imagery meditation as a manipulation check. An ANOVA revealed participants in the compassion meditation condition (*M* = 5.49, *SD* = 2.34) felt more compassionate compared to those in the focussed imagery condition (*M* = 4.26, *SD* = 2.38), *F*(1, 267) = 18.29, *p* < 0.001. Moreover, there was no significant effect of resource group on feelings of compassion, *F*(1, 267) = 0.06, *p* = 0.805, nor was there a significant interaction between resource group and condition, *F*(1, 267) = 0.42, *p* = 0.516.

#### Compassionate behavior

In total, 15.1% of participants initiated some form of sharing. A GLMM was conducted to establish the effect of resource group and condition on whether an individual initiated sharing (yes or no). Results revealed no significant effect of resource group, *X^2^*(1) = 0.56, *p* = 0.456, or condition, *X^2^*(1) = 0.29, *p* = 0.590, nor a significant interaction between the two variables, *X^2^*(1) = 0.69, *p* = 0.407. See Supplementary materials 10 for the number of times each category of sharing behavior was observed.

Altogether, individual participants initiated the sharing of 2.93 (*SD* = 12.67) LEGO bricks on average. A GLMM assessed the effect of resource group and condition on the number of LEGO bricks transferred when sharing was initiated. For this model, we used a Poisson distribution and the square root link function due to the exponential nature of the dependent variable. Results revealed no significant effect of resource group, IRR = 0.82, *p* = 0.204, or condition, IRR = 1.08, *p* = 0.647, on the number of LEGO bricks transferred when sharing was initiated. Additionally, there was no significant interaction observed between the two variables, IRR = 1.24, *p* = 0.184.

Collapsed across conditions and resource groups, participants assembled 9.35 (*SD* = 4.52) food pieces on average. The effect of condition and resource group on the number of food pieces made was assessed using an LMM. More food pieces were made by the high resource group (*M* = 10.79, *SD* = 4.93) compared to the low resource group (*M* = 7.83, *SD* = 3.46), *F*(1, 66.37) = 18.36, *p* < 0.001. However, there was no significant difference observed between the conditions and the number of food pieces assembled, *F*(1, 66.37) = 1.17, *p* = 0.284, nor was there a significant interaction between the two variables, *F*(1, 66.37) = 0.07, *p* = 0.789.

On average, the groups made approximately 6.93 (*SD* = 2.82) food pieces per minute. We assessed the effect of condition and resource group on the efficiency of LEGO brick assembly (number of pieces made by groups per minute) using a two-way ANOVA. The high resource group worked faster (*M* = 8.58, *SD* = 2.60) compared to the low resource group (*M* = 5.24, *SD* = 1.92), *F*(1, 77) = 41.83, *p* < 0.001. However, there was no significant difference in work rate based on condition, *F*(1, 77) = 0.03, *p* = 0.857, nor was there a significant interaction between condition and resource group, *F*(1, 77) = 0.08, *p* = 0.773.

#### Exploratory analyses

We conducted several exploratory Linear Mixed Models examining the effect of condition and resource group on fears of compassion as well as group dynamics and cohesion. As demonstrated in Table 2, those in the low resource group felt their groups were more cohesive (*M* = 6.99, *SD* = 2.33) relative to those in the high resource group (*M* = 5.25, *SD* = 2.29).

**Table 2:** Linear mixed models for Study 1 exploring the effect of condition and resource group on fears of compassion, as well as group dynamics and cohesion.

		Resource group		Condition		Resource group × Condition	
Outcome variable	M *(SD)*	*F*	*p*	*F*	*p*	*F*	*p*
Fears of giving compassion	20.14*(7.58)*	0.12	0.734	3.21	0.077	<0.01	0.996
Fears of receiving compassion^	17.84*(9.11)*	0.12	0.731	0.63	0.426	0.08	0.783
Feelings of competitiveness	4.92*(3.05)*	0.01	0.905	0.15	0.704	1.65	0.202
Feelings of cooperativeness	4.32*(2.77)*	0.19	0.665	3.63	0.060	0.11	0.739
“Us” versus “Them”	5.32*(2.65)*	0.34	0.562	0.09	0.760	0.02	0.896
Group cohesion	6.05*(2.46)*	22.43	<0.001***	0.30	0.588	0.03	0.854

^Indicates results from a two-way between-groups ANOVA due to singular fit warnings for LMMs. ***p < 0.001.

### Discussion

Study 1 assessed the effect of compassion meditation on working toward a shared and superordinate goal when groups have unequal resources. Overall, we found little evidence that a short-term compassion meditation resulted in greater compassionate behavior. This null effect occurred despite participants reporting feeling more compassionate after the compassion meditation relative to the focussed imagery condition. This contrasts prior work that suggests compassion meditations promote and foster compassionate actions (Trautwein et al., 2020). The finding also suggests that while brief compassion training may increase feelings of compassion, this may not translate into more compassionate behavior.

Study 1 demonstrated that a brief standalone compassion meditation did not result in greater collaboration across boundaries of groups that are unequal. It appears that this individualistic approach (i.e., where one is made to feel compassionate as an individual) may not be effective when individuals are members of groups. Here, the dynamics of the group may have a strong influence on an individual’s behavior, and interventions that target structural elements may instead be more effective. Lower inequality, for example, is thought to reduce “us” versus “them” dynamics between different resource groups (Jetten et al., 2017), potentially paving the way for greater coordinated action.

## Study 2

In line with classic social identity theorizing (Tajfel and Turner, 1979), Study 2 examined whether the structural context shapes group behavior. We proposed that structural factors, as opposed to individual factors (such as inducing individual-level compassion), may be a more important determinate of whether groups adopt superordinate goals. Previous research has shown that intergroup competition is lower when individuals or groups have more equal resources than when inequality of resources is high (Jetten et al., 2017; Sánchez-Rodríguez et al., 2018), and cooperation declines when inequality in resources is highly visible (Nishi et al., 2015). Following from this research, we explored whether lower (compared to high) inequality would result in more behavior that is in line with a superordinate goal. To examine this, we placed participants in two groups where the difference in group resources was either moderately or extremely unequal.^5^ Since lower inequality reduces competition, we expected that participants in groups that experienced moderate inequality would be more likely to act in line with a superordinate goal relative to groups in extreme inequality.^6^

### Method

Our methods and analytical approach were identical to that described in Study 1, apart from the deviations detailed below.

#### Participants and design

The sample was comprised of 173 participants (122 female, 48 male, 1 gender-diverse, 2 prefer not to say or no response) and were 20.98 years-old on average (*SD* = 4.75). Our approach to sample size and recruitment was identical to that described in Study 1. On average, participants reported having a middle-class background (*M* = 5.58, *SD* = 1.68). See Supplementary materials 2 for the full demographic description of the sample used.

The current study employed a 2 (condition: extreme inequality, moderate inequality) by 2 (resource group: high, low) between-subjects design, where participants were randomly assigned to a condition and a resource group.^7^ We were interested in the effect of these independent variables on several outcomes including compassionate behavior, fears of compassion and group cohesion.

#### Procedure

We followed an identical procedure to that described in Study 1 with a few exceptions. First, participants did not listen to an audio meditation and instead were given the task instructions immediately after giving their consent. Second, participants experienced one of two LEGO brick distributions. In line with Study 1, participants in the moderate inequality condition were in a context where the high resource group was given 500 colored and 100 non-colored bricks and the low resource group was given 100 colored and 500 non-colored bricks. We increased the magnitude of this inequality in the extreme inequality condition, where the high resource group had 560 colored and 100 non-colored bricks and the low resource group was given 40 colored and 500 non-colored bricks. In addition, those in the extreme inequality condition were given additional information about the wealth of their country (i.e., Lindithia was extremely poor and Nasherland was extremely rich; see Supplementary materials 11 for the full country descriptions for this condition).

#### Measures

All measures were identical to Study 1 (cohesion measure: *α* = 0.83, fears of giving compassion: *α* = 0.84, fears of receiving compassion: *α* = 0.89). However, we did not include the manipulation check measure that assessed how compassionate participants felt in response to the meditation.

### Results

In total, 31 experimental sessions were used for the final sample and group sizes ranged from four to nine. The full results for each analysis from Study 2 can be found in Supplementary materials 8. An independent samples *t*-test was conducted to establish whether the conditions differed in the size of the groups, and results revealed no significant difference between the extreme and moderate inequality conditions, *t*(155.42) = 0.38, *p* = 0.708. Group size was thus not considered further. The conditions and resource groups also did not differ in terms of age and gender. See Supplementary materials 9 for the means and standard deviations per condition, per resource group for each of the dependent variables.

#### Attention checks

Overall, participants were highly cognizant of the unequal resources between the two groups (*M* = 7.96, *SD* = 2.73). An LMM was conducted on the effect of resource group and condition on the extent to which the participants noticed the inequality. There were no differences between resource groups, *F*(1, 51.10) = 0.72, *p* = 0.401, or conditions, *F*(1, 51.10) = 2.26, *p* = 0.139, in the extent to which participants noticed the inequality. Likewise, there was no significant interaction between the two variables, *F*(1, 51.10) = 0.02, *p* = 0.879.

We further assessed the extent to which participants felt they had enough LEGO bricks to complete the task. An LMM was used to assess the effect of resource group and condition on this variable. A significant effect of resource group was found, *F*(1, 56.55) = 68.23, *p* < 0.001, where the high resource group (*M* = 8.34, *SD* = 2.53) felt more so than the low resource group (*M* = 3.84, *SD* = 3.06) that they had enough LEGO bricks to complete the task. However, there was no significant effect of condition, *F*(1, 56.55) = 0.002, *p* = 0.969, and no condition by resource group interaction, *F*(1, 56.55) = 2.39, *p* = 0.128.

#### Compassionate behavior

In total, 28.3% of participants initiated some form of sharing. A Generalized Linear Mixed Model (GLMM) was conducted to establish the effect of resource group and condition on whether an individual initiated sharing (yes or no). Results revealed no significant effect of resource group, *X^2^*(1) = 0.02, *p* = 0.896, or condition, *X^2^*(1) = 0.37, *p* = 0.543, nor a significant interaction between the two variables, *X^2^*(1) = 0.02, *p* = 0.878. See Supplementary materials 10 for the number of times each category of sharing behavior was observed.

**Figure 1:**
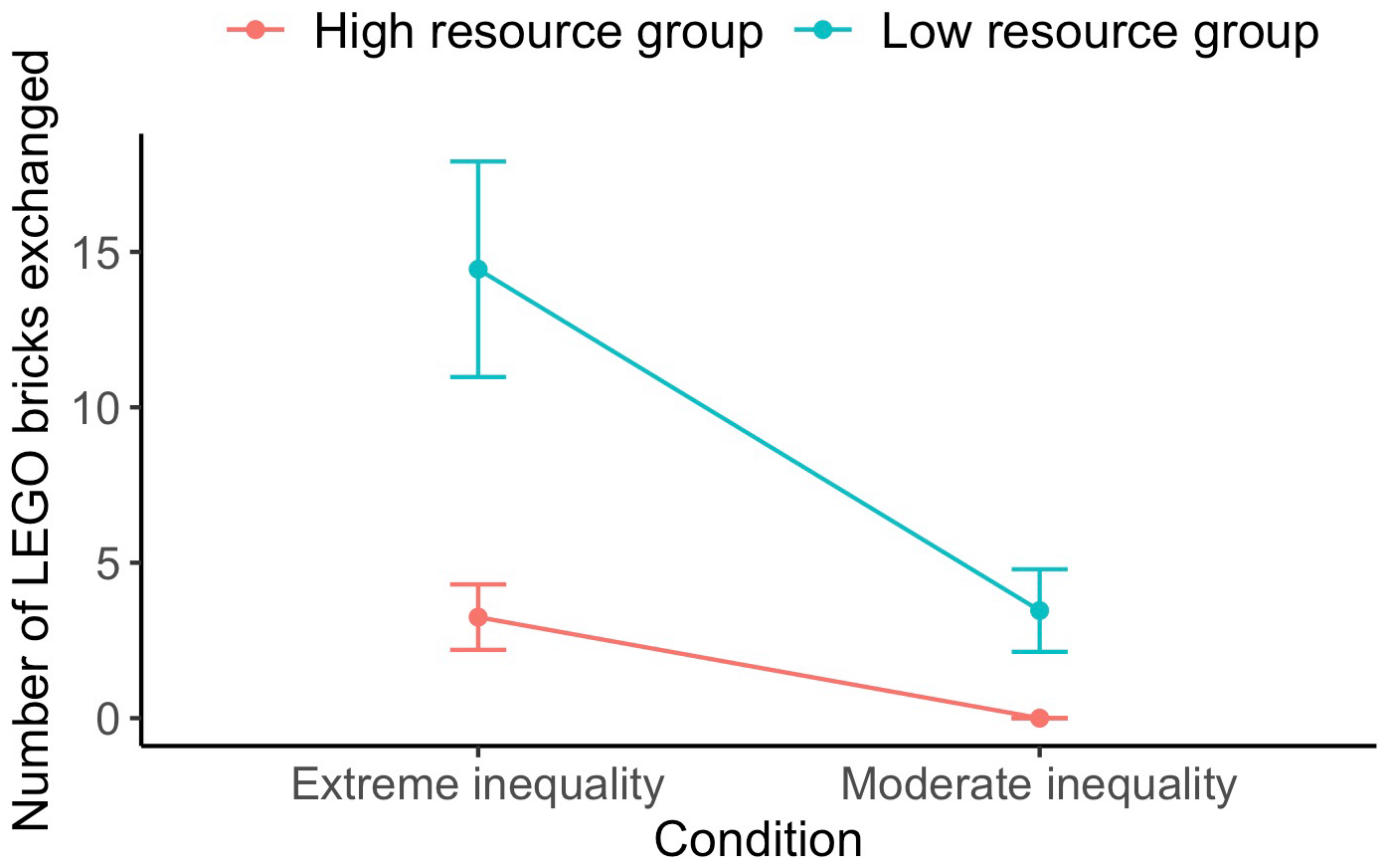
Average number of LEGO bricks transferred when sharing was initiated per condition and resource group for Study 2. Error bars represent standard errors.

Altogether, individual participants initiated the sharing of 5.27 (*SD* = 13.63) LEGO bricks on average. A GLMM assessed the effect of resource group and condition on the number of LEGO bricks transferred when sharing was initiated. For this model, we used a Poisson distribution and the square root link function due to the exponential nature of the dependent variable (see Figure 1). The low resource group (*M* = 9.22, *SD* = 18.18) initiated the sharing of more LEGO bricks compared to the high resource group (*M* = 1.71, *SD* = 5.52), IRR = 0.50, *p* = 0.004. Likewise, more LEGO bricks were transferred in instances of sharing in the extreme (*M* = 8.54, *SD* = 17.33) compared to the moderate inequality condition (*M* = 1.65, *SD* = 5.94), IRR = 1.80, *p* = 0.012. However, there was no significant interaction observed between the two variables, IRR = 0.81, *p* = 0.365.

Collapsed across conditions and resource groups, participants assembled 11.14 (*SD* = 5.65) food pieces on average. The effect of condition and resource group on the number of food pieces made was assessed using a Linear Mixed Model (LMM). More food pieces were made by the high resource group (*M* = 13.47, *SD* = 4.95) compared to the low resource group (*M* = 8.55, *SD* = 5.26), *F*(1, 53.43) = 25.89, *p* < 0.001. However, there was no significant difference observed between the conditions and the number of food pieces assembled, *F*(1, 53.43) = 3.43, *p* = 0.070, nor was there a significant interaction between the two variables, *F*(1, 53.43) = 0.04, *p* = 0.849.

On average, the groups made approximately 6.78 (*SD* = 2.41) food pieces per minute. We assessed the effect of condition and resource group on the efficiency of LEGO brick assembly (number of pieces made by groups per minute) using a two-way ANOVA. The high resource group (*M* = 8.08, *SD* = 2.32) worked faster compared to the low resource group (*M* = 5.47, *SD* = 1.69), *F*(1, 58) = 25.70, *p* < 0.001. However, there was no significant difference in work rate based on condition, *F*(1, 58) = 1.73, *p* = 0.193, nor was there a significant interaction between condition and resource group, *F*(1, 58) = 0.34, *p* = 0.561.

#### Exploratory analyses

We conducted several exploratory Linear Mixed Models examining the effect of condition and resource group on fears of compassion as well as group dynamics and cohesion. As demonstrated in Table 3, those in the low resource group (*M* = 14.84, *SD* = 8.49) reported lower fears of receiving compassion relative to those in the high resource group (*M* = 19.11, *SD* = 10.24). Those in the extreme inequality condition (*M* = 7.24, *SD* = 2.32) felt there was a greater cohesion in the resource group compared to those who experienced moderate inequality (*M* = 6.04, *SD* = 2.22).

**Table 3:** Linear mixed models for Study 2 exploring the effect of condition and resource group on fears of compassion, as well as group dynamics and cohesion.

		Resource group		Condition		Resource group × Condition	
Outcome variable	*M*(*SD*)	*F*	*p*	*F*	*p*	*F*	*p*
Fears of giving compassion	20.01*(7.24)*	3.21	0.080	<0.01	0.957	0.88	0.354
Fears of receiving compassion	17.11*(9.67)*	7.24	0.009**	0.43	0.516	0.52	0.475
Feelings of Competitiveness	5.47*(2.97)*	1.67	0.203	0.93	0.340	0.82	0.369
Feelings of Cooperativeness	4.11*(2.91)*	0.13	0.722	1.93	0.171	0.34	0.563
“Us” versus “Them”^	5.74*(2.73)*	1.41	0.237	0.25	0.616	0.59	0.444
Group cohesion	6.71*(2.35)*	0.43	0.514	6.41	0.014*	0.62	0.434

^Indicates results from a two-way between-groups ANOVA due to singular fit warnings. *p < 0.05, **p < 0.01.

### Discussion

In Study 2, we analyzed the effect of the degree of inequality on the adoption of behavior directed toward a superordinate goal. We found no consistent support for our hypothesis and results show that participants in the moderate inequality condition did not behave in line with a superordinate goal more than those in the extreme inequality condition. Overall, we found that there were no differences in whether sharing was initiated (yes or no) between conditions. However, when sharing *was* initiated in the extreme inequality condition, more LEGO bricks were involved in that transfer compared to moderate inequality. Importantly, we found no differences in the extent to which participants noticed the inequality, and both conditions yielded ceiling effects; inequality was highly salient to participants in both conditions. Low resource participants also initiated sharing more than high resource participants and reported reduced fears of receiving compassion. Moreover, our manipulation did not result in differences in feelings of competitiveness or cooperativeness. However, extreme inequality did result in greater cohesion with the group, suggesting that when differences between groups are enhanced, participants feel more united with their ingroup (Jetten et al., 2017; Jetten and Peters, 2019).

These results in combination suggest that our manipulation of the degree of inequality did not promote the adoption of compassionate behavior and attitudes. Instead, interventions that alter the normative structure, such as an informal leader promoting a pro-sharing group norm, may result in more compassionate action when groups are unequal.

## Study 3

The behavior of others around us, and in particular, the members of our ingroup, can have a dramatic effect on how we choose to act (Brown and Pehrson, 2019). In particular, highlighting norms about what individuals *should* do tends to enhance prosocial behavior (Capraro et al., 2019; Capraro and Perc, 2021; Capraro et al., 2022). We also typically favor members of our own group over members of other groups, even if the group membership is dictated by something as arbitrary as a similar colored shirt (Navarrete et al., 2012). However, when group members promote a norm that helps outgroup members, ingroup favoritism can be overridden (Reicher et al., 2006). Likewise, when participants are prompted to reflect on what they *should* do, they are less likely to favor the ingroup over the outgroup (Bilancini et al., 2020). Ambiguous situations (e.g., not knowing whether groups should compete or work together) present a particular challenge for groups (Clark and Word, 1972; Orlando, 2020). Because of this, an individual who introduces a pro-sharing group norm can become an informal leader and guide their group toward superordinate action. Past research has shown that informal leaders who offer cognitive alternatives – that is, alternatives to the current reality – can have a powerful impact on the behavior of other members (Haslam and Reicher, 2007; Zhang et al., 2013).

Study 3 aimed to explore the influence of a pro-sharing group norm on the emergence of superordinate, compassionate behavior when groups are unequal. To assess this, we utilized the same design from the moderate inequality condition in Study 2. This time, a confederate was planted in the high resource group. In our pro-sharing group norm condition, the confederate gradually prompted sharing between the groups with increasing intensity over the five-minute task period. This was compared to a control condition where the confederate instead discussed their enjoyment of LEGO bricks. While the confederate was acting as a high resource group member, they had the potential to sway the behaviour of members from both the high and low resource group. In line with prior research (Tarrant et al., 2009; Nook et al., 2016; Lay et al., 2020), we hypothesised that more compassionate behavior would be exhibited by all participants in the pro-sharing group norm condition compared to the control condition.

### Method

Our methods and analytical approach were identical to the moderate inequality condition in Study 2, with exceptions outlined below.

#### Participants and design

The sample was comprised of 160 participants (112 female, 48 male) and were 20.36 years-old on average (*SD* = 3.30). Our approach to sample size and recruitment was identical to that described in Study 1. On average, participants reported having a middle-class background (*M* = 5.59, *SD* = 1.68). See Supplementary materials 2 for the full demographic description of the sample used.

The current study employed a 2 (condition: pro-sharing group norm, control) by 2 (resource group: high, low) between-subjects design, and participants were randomly assigned to a condition and a resource group. We were interested in the effect of these independent variables on a number of outcomes including compassionate behavior, competitive sentiments, fears of compassion and group dynamics.

#### Procedure

The study followed an identical procedure to the moderate inequality condition in Study 2 with a few exceptions. Participants either experienced the implementation of a pro-sharing group norm or a control condition, and this was achieved by including a confederate in the high resource group. The confederate took on an informal leadership position in the group and spoke only during the LEGO brick assembly task. In both conditions, they spoke at one-minute intervals and were instructed to only speak to group members when spoken to. In the pro-sharing group norm condition, the prompts escalated in their intensity. The confederate first pointed out the LEGO brick inequality, then created an injunctive norm where they suggested sharing. Eventually they themselves physically shared bricks. In the control condition, the confederate spoke about their enjoyment of LEGO bricks at each minute interval. The specific prompts are outlined in Table 4. The confederate was instructed to work at a similar rate to the other group members. We chose to have a control confederate rather than a no confederate condition to control for any effects consistent discussion might have on participant behavior. That is, a confederate who speaks frequently – regardless of what they speak about – might promote a different group dynamic and this may change how participants behave.

**Table 4:** Script for the confederate across both conditions for Study 3.

	Pro-sharing group norm	Control
Prompt 1	It looks like they do not have enough LEGO bricks	I like playing with LEGO bricks
Prompt 2	Do you think we should share our LEGO bricks?	Do you like playing with LEGO bricks?
Prompt 3	I think we should share with them	It’s been a long time since I played with LEGO bricks
Prompt 4	Here, have some LEGO bricks (shares 4 LEGO bricks)	LEGO bricks are really fun

The four LEGO bricks shared by the confederate at prompt 4 were not counted in the sharing score.

#### Measures

All measures were identical to Study 2 (cohesion measure: *α* = 0.82, fears of giving compassion: *α* = 0.81, fears of receiving compassion: *α* = 0.89).

### Results

An independent samples *t*-test was conducted to examine whether the size of the groups were identical across the conditions. Results revealed a significant difference such that groups were smaller in size in the pro-sharing group norm condition (*M* = 2.36, *SD* = 0.77) compared to the control condition (*M* = 3.11, *SD* = 0.76), *t*(151) = −6.15, *p* < 0.001. This difference emerged despite careful random allocation procedures, and we thus used group size as a covariate in all analyses. The full results for each analysis from Study 3 (including the role of group size for each analysis) can be found in Supplementary materials 8. The conditions and resource groups did not differ in terms of age and gender. See Supplementary materials 9 for the means and standard deviations per condition, per resource group for each of the dependent variables.

#### Attention checks

Overall, participants were highly cognizant of the unequal resources between the two groups (*M* = 8.15, *SD* = 2.43). An LMM was conducted on the effect of resource group and condition on the extent to which the participants noticed the inequality. Those in the pro-sharing group norm condition (*M* = 8.64, *SD* = 1.92) noticed the inequality more compared to those in the control condition (*M* = 7.75, *SD* = 2.72), *F*(1, 57.69) = 6.21, *p* = 0.016. However, there were no differences between resource groups in the extent to which participants noticed the inequality, *F*(1, 63.71) = 0.80, *p* = 0.374. Likewise, there was no significant interaction between the two variables, *F*(1, 62.43) = 0.51, *p* = 0.480.

We further assessed the extent to which participants felt they had enough LEGO bricks to complete the task. An ANCOVA revealed a significant effect of resource group, *F*(1, 154) = 370.15, *p* < 0.001, where the high resource group (*M* = 9.27, *SD* = 1.62) felt they had enough LEGO bricks to complete the task more so than the low resource group (*M* = 2.84, *SD* = 2.25). However, there was no significant effect of condition, *F*(1, 154) = 2.51, *p* = 0.115, and no condition by resource group interaction, *F*(1, 154) = 0.66, *p* = 0.418.

#### Compassionate behavior

In total, 40.6% of participants initiated some form of sharing. A GLMM was conducted to establish the effect of resource group and condition on whether an individual initiated sharing (yes or no). Results revealed that participants were more likely to initiate sharing in the pro-sharing group norm condition (63.9%) compared to the control condition (21.6%), *X^2^*(1) = 18.32, *p* < 0.001. There was no significant effect of resource group, *X^2^*(1) < 0.01, *p* = 0.945, nor a significant interaction between the two variables, *X^2^*(1) < 0.01, *p* = 0.972. See Supplementary materials 10 for the number of times each category of sharing behavior was observed.

Altogether, individual participants initiated the sharing of 6.02 (*SD* = 17.08) LEGO bricks on average. A GLMM assessed the effect of resource group and condition on the number of LEGO bricks transferred when sharing was initiated. For this model, we used a Poisson distribution and the square root link function due to the exponential nature of the dependent variable. More LEGO bricks were transferred in instances of sharing in the pro-sharing group norm condition (*M* = 11.00, *SD* = 22.92) compared to the control condition (*M* = 1.94, *SD* = 8.20), IRR = 2.88, *p* < 0.001. Results additionally revealed no significant effect of resource group, IRR = 1.26, *p* = 0.375, and there was no significant interaction observed between the two variables, IRR = 1.02, *p* = 0.926.

**Figure 2:**
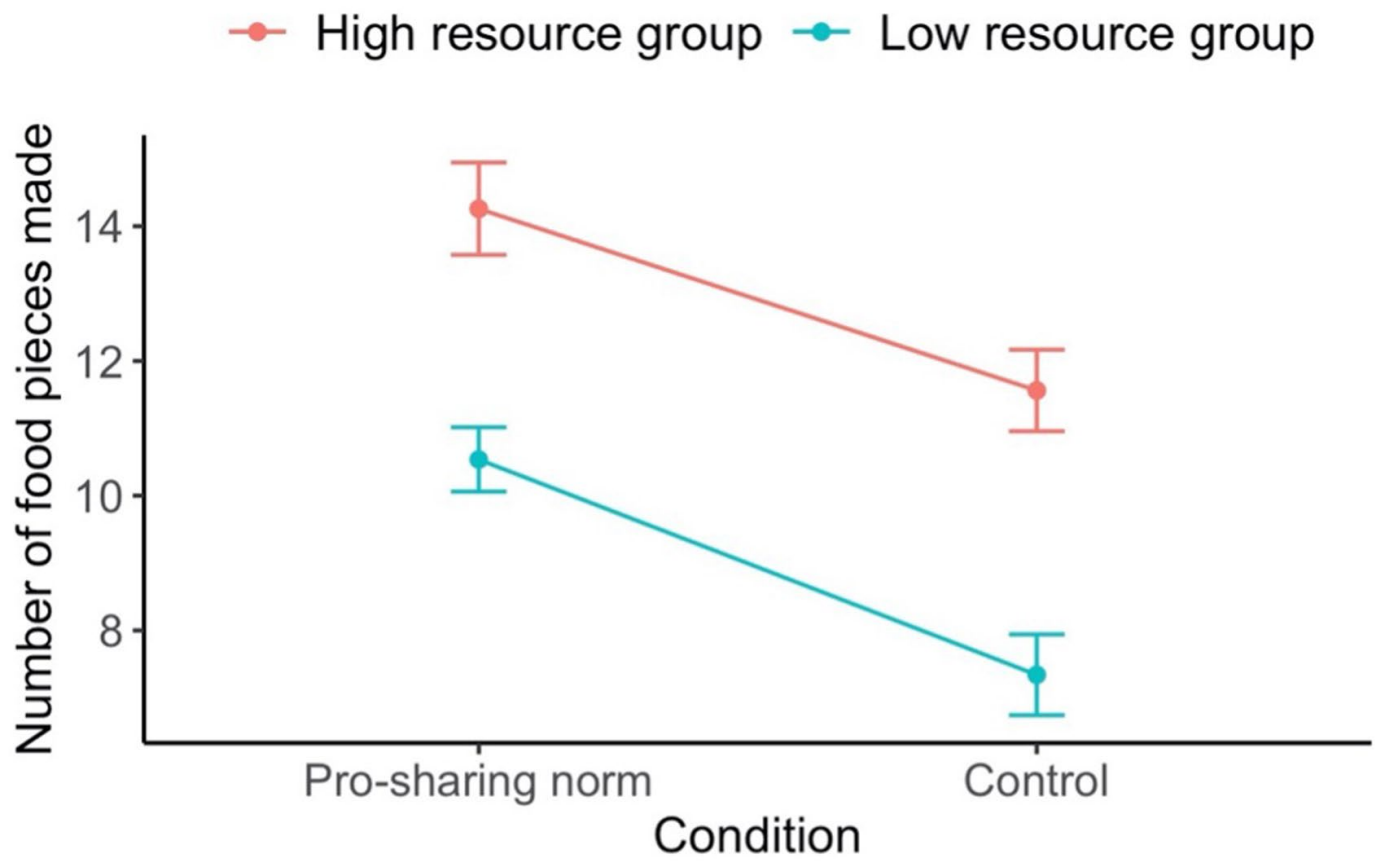
Number of food pieces made by individuals on average per condition and resource group for Study 3. Error bars represent standard errors.

Collapsed across conditions and resource groups, participants assembled 10.58 (*SD* = 4.44) food pieces on average. The effect of condition and resource group on the number of food pieces made was assessed using an LMM (see Figure 2). More food pieces were made by the high resource group (*M* = 12.72, *SD* = 4.05) compared to the low resource group (*M* = 8.83, *SD* = 3.98), *F*(1, 59.36) = 18.78, *p* < 0.001. Furthermore, more food pieces were assembled in the pro-sharing group norm condition (*M* = 12.14, *SD* = 3.85) compared to the control condition (*M* = 9.31, *SD* = 4.51), *F*(1, 57.46) = 6.30, *p* = 0.015. However, there was no significant interaction between the two variables, *F*(1, 60.41) = 0.19, *p* = 0.661.

On average, the groups made approximately 6.53 (*SD* = 2.36) food pieces per minute. We assessed the effect of condition and resource group on the efficiency of LEGO brick assembly (number of pieces made by groups per minute) using a two-way ANCOVA. The high resource group worked faster (*M* = 8.08, *SD* = 2.08) compared to the low resource group (*M* = 4.97, *SD* = 1.42), *F*(1, 59) = 88.50, *p* < 0.001. Those in the pro-sharing group norm condition (*M* = 6.52, *SD* = 2.24) were also more efficient at making food relative to those in the control condition (*M* = 6.54, *SD* = 2.53), *F*(1, 59) = 5.96, *p* = 0.018. Accounting for group size, the estimated marginal mean for efficiency in food assembly was higher for the pro-sharing group norm condition (*M* = 7.04, *SE* = 0.28) compared to the control condition (*M* = 5.95, *SE* = 0.30). However, there was no significant interaction between condition and resource group, *F*(1, 59) = 1.36, *p* = 0.248.

#### Exploratory analyses

**Figure 3:**
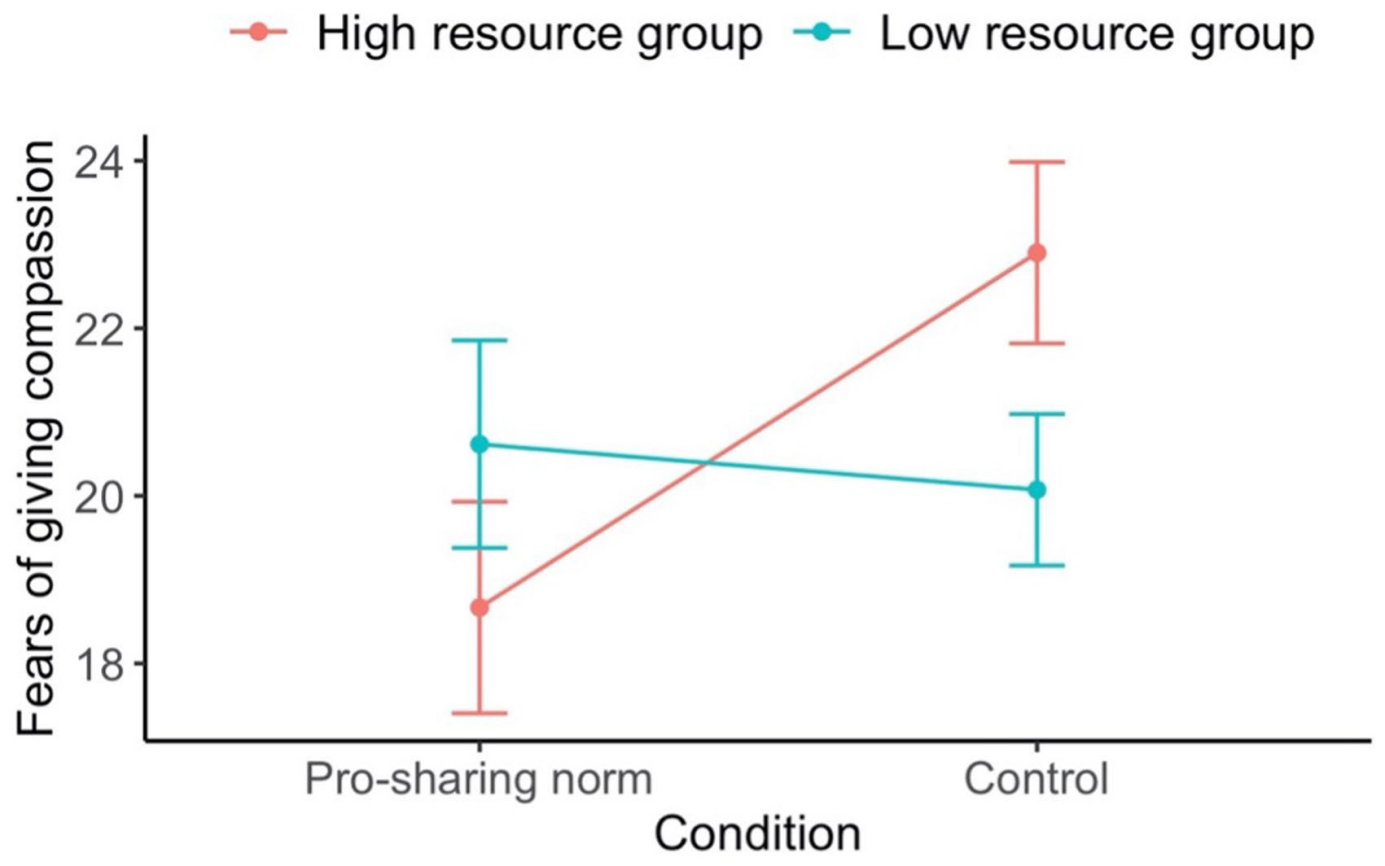
Average fears of giving compassion per condition and resource group for Study 3. Higher values indicate greater fears of giving compassion. Error bars represent standard errors.

We conducted several exploratory Linear Mixed Models examining the effect of condition and resource group on fears of compassion as well as group dynamics and cohesion. As demonstrated in Table 5, there was a significant interaction between resource groups and conditions on fears of giving compassion (see Figure 3). Follow up simple effect analyses revealed a significant effect for the high resource group only, *F*(1, 55.3) = 7.64, *p* = 0.008, such that the high resource group members in the compassionate norm condition (*M* = 18.67, *SD* = 7.25) had reduced fears of being compassionate compared to high resource group members in the control condition (*M* = 22.90, *SD* = 6.84). Moreover, those in the low resource group (*M* = 6.00, *SD* = 2.76) felt more like the context was competitive compared to those in the high resource group (*M* = 4.53, *SD* = 2.85). Participants in the pro-sharing group norm condition (*M* = 6.21, *SD* = 2.64) felt the context was more cooperative compared to those in the control condition (*M* = 3.56, *SD* = 2.68). Likewise, participants in the pro-sharing group norm condition (*M* = 4.93, *SD* = 2.49) felt the context was less one of “us” versus “them” compared to those in the control condition (*M* = 6.06, *SD* = 2.83).

**Table 5:** Linear mixed models for Study 3 exploring the effect of condition and resource group on fears of compassion, as well as group dynamics and cohesion.

		Resource group		Condition		Resource group × Condition	
Outcome variable	M*(SD)*	*F*	*p*	*F*	*p*	*F*	*p*
Fears of giving compassion	20.64*(7.02)*	<0.01	0.960	3.54	0.066	4.37	0.041*
Fears of receiving compassion	18.02*(9.86)*	0.12	0.734	0.88	0.353	0.85	0.360
Feelings of Competitiveness^	5.32*(2.89)*	10.30	0.002**	1.84	0.177	3.55	0.061
Feelings of Cooperativeness	4.75*(2.97)*	0.03	0.866	19.42	<0.001***	0.01	0.922
“Us” versus “Them”	5.55*(2.73)*	3.68	0.060	5.29	0.025*	2.63	0.110
Group cohesion	6.33*(2.20)*	0.10	0.758	<0.01	0.957	0.46	0.501

^Indicates results from a two-way between-groups ANCOVA due to singular fit warnings. *p < 0.05, **p < 0.01, ***p < 0.001.

### Discussion

Study 3 revealed the effect of a confederate who took on an informal leadership role in the group and changed the status quo by introducing a pro-sharing group norm. We found that a fellow group member who promotes sharing can have a significant influence on the behavior of other groups members—participants were more likely to initiate sharing, transferred more LEGO bricks between the groups, worked faster and made more food pieces when a confederate suggested sharing. Importantly, participants in this condition felt that the context was more cooperative and less one of ‘us’ vs. ‘them’, and the high resource participants had reduced fears of being compassionate. Together, these findings suggest that a member who imbeds a pro-sharing group norm within a group can have a powerful influence on the behavior and mentality of other group members—their leadership behavior decreases intergroup competition, and compassionate action can be achieved.

## General discussion

When Covid-19 surged, wealthy countries hoarded vaccines, and poorer countries – who were not only battling poverty but also the devastating impact of the virus – were left without (Rouw et al., 2021). How do we promote compassionate action under these conditions? Here we aimed to understand effective ways of orienting individuals toward a superordinate, compassionate goal when they were embedded in unequal groups, *via* an individualistic intervention (Study 1: compassion meditation training), structural intervention (Study 2: altering inequality), and a normative intervention promoting a different way to respond to the status quo (Study 3: pro-sharing group norm). We found little evidence that compassion meditation and varying the degree of inequality enhanced the adoption of compassionate action. However, when a confederate took the lead by introducing a pro-sharing group norm, participants engaged in more compassionate behavior and adopted a collaborative approach to the task.

The introduction of a pro-sharing group norm resulted in enhanced compassionate action and attitudes, and this finding is in line with past work suggesting norms can have a significant shift on group behavior (Sherif, 1958; Gaertner et al., 2000; Reicher et al., 2006; Tarrant et al., 2009; Nook et al., 2016; DiBenigno, 2018; Lay et al., 2020; Martinez-Ebers et al., 2021). Additionally, past research shows that individuals who offer a cognitive alternative to the current status quo can become informal leaders and sway the behavior of their group (Haslam and Reicher, 2007). Here participants transferred more LEGO bricks between the groups, interpreted the task as cooperative, worked faster and, critically, created more food for ‘starving people’. Moreover, the high resource group had reduced fears of being compassionate relative to the control condition, suggesting that the introduction of a pro-sharing group norm paved the way for group members to feel more positive about behaving compassionately toward others. It remains unclear however if the groups would adopt superordinate behavior if the confederate was instead a member of the low resource group, and this is a promising direction for future research. It is also unclear whether the confederate introduced a norm of sharing as intended or whether their comments drew attention to the unequal resources, and this instead prompted sharing. While participants reported noticing the inequality more when the confederate introduced the sharing norm, they were still highly aware of the inequality in the control condition. Nonetheless, future research should include questions about how participants view the norms of the group and assess whether this altered by condition.

On the other hand, our individualistic intervention – a compassion meditation – did not promote compassionate action or attitudes. While participants reported feeling more compassionate, there was no evidence that this translated to behavior – a phenomenon that is in line with past research demonstrating a gap between attitudes and behavior (Blake et al., 2014; Liu et al., 2016). This further suggests that while compassion meditations may alter attitudes (Chwyl et al., 2021) and behavior in some settings (Condon et al., 2013), such interventions may be too individualistic to affect compassion in a group setting. However, we only assessed the effect of short-term interventions and longer-term interventions may instead prove fruitful. Likewise, our structural intervention – varying the degree of inequality between the groups – also did not result in any meaningful changes in compassionate actions or attitudes. While enhanced inequality did lead to more LEGO bricks being transferred between the groups (likely in response to a clearer need for more LEGO bricks), this did not result in more food pieces being made. This intervention also did not impact attitudes, and this may have been because the situation invoked two competing motivations; while the need for sharing was more tangible under extreme inequality, unequal resources (whether extreme or slightly less so) suppress compassionate action (Côté et al., 2015; Sands, 2017; Kirkland et al., 2020).

Together, these three studies have revealed several insights about human behavior in a previously unexplored context. We assessed the effect of three different interventions from diverse literatures to establish which approach is most effective. The efficacy of these interventions was measured across a variety of behavioral and self-report outcomes, giving us greater certainty of the effects. In addition to theoretical contributions, these studies also have significant practical applications. In a world of increasingly complex social dilemmas, there have been growing discussions about how to promote a more compassionate world – for example by getting rich countries to assist poor countries in their acquisition of Covid-19 vaccinations (Rouw et al., 2021). Our work suggests that leadership by one individual, whether it be an individual person or possibly an individual country, may set a norm that can have a positive domino effect on compassionate actions more broadly.

### Limitations and future research

Despite these strengths, our work has produced several questions that warrant future research. While our experimental approach allowed us to gain a high degree of control, the assembly of LEGO bricks is distantly related to the acquisition of real-world resources. Thus, future work is needed to assess effects of these kinds of manipulations in real-world settings. Additionally, the endowments were windfall gains, and people tend to be less generous with resources when they are instead earned (Carlsson et al., 2013; Li et al., 2019). To test this possibility, future work should compare the effect of windfall versus earned resources on intergroup interactions in this context. Moreover, future work may wish to also vary the degree of inequality within-groups (e.g., by providing individuals within the same group with differing numbers of LEGO bricks) and explore how this interacts with between-group inequality.

We have also defined compassion as any action that aims to maximize the food creation for ‘starving people’ and placed behavior such as sharing and food assembly under this definition. However, this may not be the only motivation that is driving participants to engage in sharing and food creation. For example, high resource participants may feel pity or awkwardness directed toward the low resource group due to their lack of LEGO bricks. Future work should directly assess the motivations that drive participant sharing behavior and establish whether these are compassionate in origin. We also did not directly compare interventions across studies, and future research may wish to test which manipulations yield the largest effect size. Finally, participants were disproportionately female, largely comprised of first-year students and came from a W.E.I.R.D. population (Western, Educated, Industrialized, Rich and Democratic). Future work should extend upon these findings in more representative and culturally diverse samples.

### In conclusion

The human capacity for compassion is one of our most extraordinary traits, yet we do not always help those who are suffering. Here we aimed to establish how to foster compassionate action and promote the adoption of a superordinate goal under situations of group inequality. We assessed the effect of three interventions: compassion meditation, altering the degree of inequality, and implementing a pro-sharing group norm. Compassion meditation and changing the degree of inequality had no meaningful effect on compassionate action. The introduction of a pro-sharing group norm instead had a marked influence on the behavior and attitudes of the unequal groups. This work offers new insights into the feasibility of different interventions to foster compassionate behavior, which may be critical in promoting a more unified world.

## Data availability statement

The datasets presented in this study can be found in online repositories. The names of the repository/repositories and accession number(s) can be found at: Open Science Framework: https://osf.io/fjp6b/?view_only=b51cca6755654a76b8d1c8d77a3cfa53.

## Ethics statement

The studies involving human participants were reviewed and approved by Human Research Ethics Committee at the University of Queensland (protocol number: 2018002500). The participants provided their written informed consent to participate in this study.

## Author contributions

KK, JJ, MW, and JK designed the experiments and provided feedback on the manuscript. JK and KK supervised data collection. KK conducted analyses and interpreted the results. All authors contributed to the article and approved the submitted version.

## Funding

This research as well as the open access fee was supported by an Australian Research Council Laureate Fellowship (FL180100094) awarded to JJ.

## Conflict of interest

The authors declare that the research was conducted in the absence of any commercial or financial relationships that could be construed as a potential conflict of interest.

## Publisher’s note

All claims expressed in this article are solely those of the authors and do not necessarily represent those of their affiliated organizations, or those of the publisher, the editors and the reviewers. Any product that may be evaluated in this article, or claim that may be made by its manufacturer, is not guaranteed or endorsed by the publisher.

## Author Disclaimer

LEGO®, the LEGO® logo, the Brick and Knob configuration and the MINIFIGURE figurine are trademarks and/or copyrights of the LEGO Group of Companies, which does not sponsor, authorize or endorse this article.

## Footnotes

^1^We pre-registered additional hypotheses regarding a dependent variable: entitled behavior as measured by more taking of sweets (Piff et al., 2012). However, this variable falls outside our primary research concerns and we have elected not to include any information about it here. However, descriptions of this variable and findings have been included in Supplemental materials 1.

^2^We only analyzed the variables from the survey that were central to our primary aims. However, the full survey can be located on OSF.

^3^Because we were not able to ascertain the efficiency for each individual (due to practical constraints with the video recorded footage), we have reported the overall efficiency of the resource group.

^4^For all three studies, ANOVAS were conducted where LMMs produced ICC values that were indistinguishable from zero.

^5^To test this, we heightened inequality relative to Study 1 (rather than lowered inequality). This decision was made because, in our paradigm, groups that possess equal resources would have no superordinate reason to collaborate. Hence, heightening the differences between groups is the only feasible way to test the role of inequality in this paradigm.

^6^We originally preregistered a null hypothesis by mistake (i.e., that there would be no difference between groups), and believe this does not accurately reflect the body of research existing at the time. We have changed our hypothesis to better reflect the state of the literature prior to creating this study.

^7^It is worth noting that this manipulation creates a natural confound. That is, when the inequality changes between the groups (i.e., moderate versus extreme), we also change the level of resources each group has; the low resource group in moderate inequality has more resources than the low resource group in extreme inequality. However, this is unavoidable as manipulating the gap between the low and the high resource group also varies the absolute amount of wealth each group has.
