# Supplementary material for: Promoting prosocial behavior in an unequal world
Source: Front Psychol. 2023 Feb 2;13:1021093. doi: 10.3389/fpsyg.2022.1021093 (PMC9932976; doi:10.3389/fpsyg.2022.1021093)
Supplement: Supplementary file 1 [file Data_Sheet_1.docx]

# Supplementary Materials 1 – Findings for Entitled Behaviour

We originally preregistered a second hypothesis for each study looking at the effect of condition on entitled behaviour (Piff et al., 2012). However, this measure was peripheral to the focus of the study, and we have included the results here instead. At the conclusion of the LEGO brick assembly task, a box of sweets were placed on the table. As a measure of entitled behaviour, we recorded the number of sweets taken by each individual.

**Study 1 – Compassion Meditation**

Table 1

Linear Mixed Model for the Effect of Condition and Resource Group on Entitled Behaviour

|  | **Number of Sweets Taken** | | |
| --- | --- | --- | --- |
| *Predictors* | *Incidence Rate Ratios* | *CI* | *p* |
| (Intercept) | 1.99 | 1.73, 2.28 | <.001 |
| Condition | 0.94 | 0.82, 1.08 | .393 |
| Resource | 1.03 | 0.90, 1.18 | .674 |
| Condition : Resource | 1.00 | 0.87, 1.15 | .999 |
| **Random Effects** | | | |
| Residual | 0.25 | | |
| Group (intercept) | 0.22 | | |
| ICC | .46 | | |
| N_Group_ | 65 | | |
| Observations | 233 | | |
| Marginal R^2^ / Conditional R^2^ | .009 / .469 | | |

**Study 2 – Inequality**

Table 2

Linear Mixed Model for the Effect of Condition and Resource Group on Entitled Behaviour

|  | **Number of Sweets Taken** | | |
| --- | --- | --- | --- |
| *Predictors* | *Incidence Rate Ratios* | *CI* | *p* |
| (Intercept) | 2.08 | 1.82, 2.36 | <.001 |
| Condition | 0.97 | 0.85, 1.10 | .641 |
| Resource | 1.02 | 0.90, 1.16 | .718 |
| Condition : Resource | 0.97 | 0.85, 1.10 | .601 |
| **Random Effects** | | | |
| Residual | 0.25 | | |
| τ_00_ _Group_ | 0.14 | | |
| ICC | .37 | | |
| N _Group_ | 62 | | |
| Observations | 173 | | |
| Marginal R^2^ / Conditional R^2^ | .007 / .371 | | |

**Study 3 – Superordinate Norm**

Table 3

Linear Mixed Model for the Effect of Condition and Resource Group on Entitled Behaviour

|  | **Number of Sweets Taken** | | |
| --- | --- | --- | --- |
| *Predictors* | *Incidence Rate Ratios* | *CI* | *p* |
| (Intercept) | 2.02 | 1.76, 2.32 | <.001 |
| Condition | 0.99 | 0.87, 1.13 | .919 |
| Resource | 0.98 | 0.86, 1.12 | .736 |
| Condition : Resource | 0.99 | 0.87, 1.13 | .917 |
| **Random Effects** | | | |
| Residual | 0.25 | | |
| τ_00_ _Group_ | 0.12 | | |
| ICC | .32 | | |
| N _Group_ | 54 | | |
| Observations | 138 | | |
| Marginal R^2^ / Conditional R^2^ | .001 / .322 | | |

# Supplementary Materials 2 – Demographic Information

**Study 1**

Table 4

Demographic Statistics for the Sample

| **Demographic Variable** | **N** | **%** |
| --- | --- | --- |
| Education |  |  |
| Year 10 or equivalent | 2 | 0.7 |
| Year 12 or equivalent | 150 | 53.4 |
| Certificate/diploma | 48 | 17.1 |
| University (undergraduate) degree | 70 | 24.9 |
| Higher education degree (graduate diploma) | 4 | 1.4 |
| Postgraduate degree (Masters/PhD) | 7 | 2.5 |
| Ethnicity |  |  |
| Australian | 89 | 31.6 |
| Indigenous Australian | 1 | 0.4 |
| European | 10 | 3.5 |
| Sub-Saharan African | 1 | 0.4 |
| Middle Eastern | 4 | 1.4 |
| South-East Asian | 103 | 36.5 |
| North-East Asian | 13 | 4.6 |
| Southern Asian | 16 | 5.7 |
| Central Asian | 11 | 3.9 |
| People of the Americas | 1 | 0.4 |
| Other | 2 | 0.7 |
| Multiple ethnicities | 31 | 11.0 |
| Income |  |  |
| 0 – 10,000 | 203 | 72.2 |
| 10,000 – 20,000 | 45 | 16.0 |
| 20,000 – 30,000 | 15 | 5.3 |
| 30,000 – 40,000 | 9 | 3.2 |
| 40,000 – 50,000 | 3 | 1.1 |
| 50,000 – 60,000 | 2 | 0.7 |
| 60,000 – 70,000 | 3 | 1.1 |
| 70,000 – 80,000 | 1 | 0.4 |

*Note:* Income is in $AUD

**Study 2**

Table 5

Demographic Statistics for the Sample

| **Demographic Variable** | **N** | **%** |
| --- | --- | --- |
| Education |  |  |
| Year 10 or equivalent | 2 | 1.2 |
| Year 12 or equivalent | 99 | 57.2 |
| Certificate/diploma | 24 | 13.9 |
| University (undergraduate) degree | 34 | 19.7 |
| Higher education degree (graduate diploma) | 4 | 2.3 |
| Postgraduate degree (Masters/PhD)  No response | 3  7 | 1.7  4.0 |
| Ethnicity |  |  |
| Australian | 75 | 43.4 |
| New Zealand | 3 | 1.7 |
| European | 5 | 2.9 |
| Sub-Saharan African | 1 | 0.6 |
| Middle Eastern | 1 | 0.6 |
| South-East Asian | 44 | 25.4 |
| North-East Asian | 6 | 3.5 |
| Southern Asian | 4 | 2.3 |
| Central Asian | 5 | 2.9 |
| People of the Americas | 2 | 1.2 |
| Other | 4 | 2.3 |
| Multiple ethnicities | 22 | 12.7 |
| No response | 1 | 0.6 |
| Income |  |  |
| 0 – 10,000 | 107 | 61.8 |
| 10,000 – 20,000 | 36 | 20.8 |
| 20,000 – 30,000 | 15 | 8.7 |
| 30,000 – 40,000 | 6 | 3.5 |
| 40,000 – 50,000 | 1 | 0.6 |
| 50,000 – 60,000 | 1 | 0.6 |
| 60,000 – 70,000 | 1 | 0.6 |
| 70,000 – 80,000 | 1 | 0.6 |
| 80,000 – 90,000 | 1 | 0.6 |
| 90,000 – 100,000 | 1 | 0.6 |
| 100,000 + | 3 | 1.7 |

*Note:* Income is in $AUD.

**Study 3**

Table 6

Demographic Statistics for the Sample

| **Demographic Variable** | **N** | **%** |
| --- | --- | --- |
| Education |  |  |
| Year 10 or equivalent | 1 | 0.6 |
| Year 12 or equivalent | 109 | 68.1 |
| Certificate/diploma | 14 | 8.8 |
| University (undergraduate) degree | 26 | 16.3 |
| Higher education degree (graduate diploma) | 2 | 1.3 |
| Postgraduate degree (Masters/PhD) | 8 | 5.0 |
| Ethnicity |  |  |
| Australian | 72 | 45.0 |
| European | 3 | 1.9 |
| Sub-Saharan African | 1 | 0.6 |
| Middle Eastern | 2 | 1.3 |
| South-East Asian | 35 | 21.9 |
| North-East Asian | 4 | 2.5 |
| Southern Asian | 6 | 3.8 |
| Central Asian | 6 | 3.8 |
| People of the Americas | 3 | 1.9 |
| Other | 6 | 3.8 |
| Multiple ethnicities | 22 | 13.8 |
| Income |  |  |
| 0 – 10,000 | 110 | 68.8 |
| 10,000 – 20,000 | 27 | 16.9 |
| 20,000 – 30,000 | 9 | 5.6 |
| 30,000 – 40,000 | 1 | 0.6 |
| 40,000 – 50,000 | 6 | 3.8 |
| 50,000 – 60,000 | 2 | 1.3 |
| 60,000 – 70,000 | 2 | 1.3 |
| 90,000 – 100,000 | 3 | 1.9 |

*Note:* Income is in $AUD

# Supplementary Materials 3 – Randomization Procedure

**Condition Randomization**

Sessions were randomized to a condition using a randomized number generator (https://www.graphpad.com/quickcalcs/randomN1.cfm).

**Resource Group Randomization**

Before participants entered the experiment room, they were instructed to randomly choose a token from an opaque bag. This token was either black or white and they were instructed to sit on the side of the table with either black or white pieces of paper stuck to the chairs. Participants who drew the white token were assigned to the low resource group and participants who drew the black token were assigned to the high resource group. The number of tokens was always matched to the size of the group prior to participants entering the experimental room. In the event of odd numbers in Study 1 and Study 2, an extra high resource (black) token was added to the bag, meaning the high resource group had more participants than the low resource group. The process differed slightly in Study 3 due to the addition of a confederate. The confederate was always added to the high resource group. When there were odd numbers of participants in Study 3, one high resource (black) token was placed in the bag. In the event of even numbers in Study 3, the number of high (black) and low (white) tokens were even.

# Supplementary Materials 4 – Study 1 Meditation Scripts

**Condition 1 – Compassion Meditation**

Hi and welcome to this audio exercise on compassion. My name is James, and I’ll be guiding you through the exercise. During this exercise I’ll ask you to focus on your posture and breathing, and these exercises will help us slow down a little bit and to start to activate our compassionate motivation. All up this will take about 5-7 minutes to complete. And all you need to do is follow my instructions as we go along.

So get comfortable in your chair, feet flat on the floor, sitting straight, shoulders back and chest open, and if you are comfortable gently close our eyes of if you prefer just direct your gaze downwards.

To begin just focus on your breathing, noticing the air coming in through your nose, down into your diaphragm area, staying a short while and then moving back out through your nose. Notice how your diaphragm moves gently as you breathe in and out.

And now we are going to move into a style of breathing called soothing rhythm breathing. This style of breathing is focused on having a smoothness of breath. So when you breathe out just let it be a gentle smooth exhale rather than anything forced – so you are gently letting air out, smoothly. And the same for the inhale, you are breathing the air in smoothly. Not rushed and then hold, but slowly breathing in.

And we are going to do this for a count of breathing in for 5 seconds and out for 5 seconds. So I will count you into this by saying in 2,3 out 2,3; in 2,3,4 out 2,3,4; and in 2,3,4,5 and out, 2,3,4,5. And when you breathe try to notice the slight pause at the top and the bottom of the breath.

So let’s begin. In 2,3 out 2,3; in 2,3,4 out 2,3,4; and in 2,3,4,5 and out, 2,3,4,5.

And keep going with that rhythm for the next minute or so.

As you develop your rhythm, notice and focus on the feeling of inner slowing with each out breath. Say on each alternate out breath a friendly supportive voice

*Body Slowing Down*

*Mind Slowing Down*

When you say these words say them slowly.

Now as we are doing this your mind will most likely wander off, and when it does try to notice that and then gently bring your attention back to the breath. You might think, “*am I doing this right?*” or feel slightly irritated or frustrated with exercise. If so, just note that, and using your friendly inner voice tone return to the soothing rhythm breathing.

Breathing steadily in and out; just noticing the flow of air coming in and out of your nose.

A sense of the weight of your body resting in the chair and the floor underneath – you may feel slightly heavier in the chair now – allow yourself to feel held and supported. So feeling the stableness in your body that has come from slowing your breathing and feeling slightly heavier. Imagine sitting like a mountain, strong and stable.

So now that we are in this position of groundedness and stability we are now going to start to develop, as best we can, the mind and body pattern of what we call the compassionate-self.

To begin, you might like to imagine, as best you can, that you are a wise and compassionate person. It may be useful to bring to mind a situation when you felt compassion for another person. Try to remember what you thought, your feelings of compassion and care and your genuine wish for that person to be free of suffering.

Just notice what you were doing to show compassion, and how you were doing things, what you were saying and how you are saying things to help this person. Try and notice what qualities were present when you were showing compassion.

So compassion is simply, “*a sensitivity to suffering in self and others with a commitment to try to alleviate and prevent it*”.

So at the heart of compassion is courage. That is when we become sensitive and aware of suffering, pain, or distress, compassion requires us to move towards the thing which is distressing, not away. It can sometimes be scary to move towards suffering, so that we can try to take steps or actions to be compassionate to help others, so that is why courage is so important to compassionate action.

Compassion also has this deep wisdom about it. Compassion is focused in how you can be helpful rather than hurtful or harmful. So compassion requires a deep wisdom to try to work out what might be helpful to reduce or prevent suffering.

Finally, compassion is committed to trying to be helpful, even when these things might be effortful, compassion has this commitment this desire to be helpful to people, and to use wisdom and courage where you can to help others as well as yourself.

So at its core compassion is focused on:

How can I be helpful and not harmful or hurtful.

Try to really focus on this, what it feels like to have this intention of wanting to be helpful to yourself and to others.

And now, I’d just like you to imagine, you have become this ideal compassionate person. How would you engage with the world?

How would you respond to yourself when in pain or struggling? How would you respond to others when they are suffering? What would you say, how would you say it? Try to get a feel for what it would be like, to respond as your compassionate-self. It might be tricky, but just try to imagine, as best you can.

All we are doing is trying to get a feel for what it would be like, to be this compassionate person.

Now just letting that imagery go and return slowly back to our breath, noticing the rise and fall of our belly, and noticing the contact your body makes with the chair. Getting a greater sense of the room and space around us.

And see if you can take this compassionate motivation into your daily living.

And when you hear the bells just follow the sound for as long as you can and then open your eyes.

Thank you.

**Condition 2 – Focussed Imagery Meditation**

Hi, I’m James, and today I will take you through an exercise called a Focused Imagery technique. The aim of the technique will be to visualize different parts of your face, arms, and legs, and that of other people’s face, arms and legs. All up it will take about 5-7 minutes to complete. And all you need to do is follow my instructions as we go along

So to begin with get yourself comfortable. Sit reasonably upright, but not overly rigid. Rest your hands by your side or on your legs. And when you are ready gently close your eyes

***Focused Imagery Towards Self***

And when you are ready I would like for you to bring to mind and visualize different parts of your face. Just create an image of your face in your mind’s eye.

And now that you have created that image of your face in your mind’s eye, all I want you to do now is to focus on the shape of your face. Imagine scanning the contours of the face, from the bottom of your chin, to the top of your forehead.

And now I’d like you to change your focus to your eyebrows. Notice the colour of your eyebrows and really visualise them. And now just imagine scanning the outline of your eyebrows, from the inside to the outside.

Alright, now if you could change your focus to your eyes. Visualize the various shades of colour in your eyes, from the outside of your eyes to the inside of your eyes.

Now if you could change your focus to your ears. Imagine scanning the contours of your left ear. Then, imagine scanning the contours of your right ear. Visualize what they look like in your mind.

Now I’d like you to focus on your nose. Create an image of your nose in your mind’s eye. And now I’d like you to imagine drawing a line right down the middle of your nose, right from the top to the base of your nose.

And finally, now I’d like you to focus on your lips. Imagine scanning the outline of your lips. Visualize their colour and texture.

***Focused Imagery Towards a Friend***

OK now you can let go of that visualization, and now I’d like you to bring to mind an image of one of your friends, the one that first comes to mind.

And when you are ready I would like for you to bring to mind and visualize different parts of his or her face. Just create an image of their face in your mind’s eye.

And now that you have created that image of their face in your mind’s eye, all I want you to do now is to focus on the shape of their face. Imagine scanning the contours of their face, from the bottom of their chin, to the top of their forehead.

And now I’d like you to change your focus to their eyebrows. Notice the colour of your friend’s eyebrows and really visualise them. And now just imagine scanning the outline of their eyebrows, from the inside to the outside.

Alright, now if you could change your focus to their eyes. Visualize the various shades of colour in their eyes, from the outside of their eyes to the inside of their eyes.

Now if you could change your focus to their ears. Imagine scanning the contours of their left ear. Then, imagine scanning the contours of their right ear. Visualize what your friends ears look like in your mind.

Now I’d like you to focus on their nose. Create an image of their nose in your mind’s eye. And now I’d like you to imagine drawing a line right down the middle of their nose, right from the top to the base of their nose.

And finally, now I’d like you to focus on their lips. Imagine scanning the outline of their lips. Visualize their colour and texture.

***Focused Imagery Towards Self***

OK now you can let go of that visualization, and now I’d like you to bring to mind an image of yourself again. And when you are ready I would like for you to visualize different parts of your body. Just create an image of your whole body in your mind’s eye.

I’d like you to first focus on your hands. Notice the shape of your hands in your mind’s eye. Imagine scanning the outlines of your hands, around each finger, one by one. Really visualising what they look like, noticing the wrinkles around the knuckles.

Now extending from this I’d like you to focus on the shape of your arms. Imagine scanning the contours of the arms, from the base of your wrists, then move up to the forearms, all the way up to your shoulders. Start with the left arm, and then move to the right arm. Slowly visualising each part of the arm.

Now I’d like you to change your focus to your legs. Imagine scanning the outline of your legs, from the base of your feet, around your toes, all the way up to the top of your legs. Start with the left leg, and then move to the right leg. Slowly visualising each part of the leg.

***Focused Imagery Towards a Friend***

OK now you can let go of that visualization, and now I’d like you to bring to mind an image of a friend, the one that first comes to mind.

And when you are ready I would like for you to visualize different parts of his or her body. Just create an image of their whole body in your mind’s eye.

I’d like you to first focus on their hands. Notice the shape of their hands in your mind’s eye. Imagine scanning the outlines of their hands, around each finger, one by one. Really visualising what they look like, noticing the wrinkles around the knuckles.

Now extending from this I’d like you to focus on the shape of their arms. Imagine scanning the contours of the arms, from the base of their wrists, then move up to the forearms, all the way up to their shoulders. Start with the left arm, and then move to the right arm. Slowly visualising each part of the arm.

Now I’d like you to change your focus to their legs. Imagine scanning the outline of their legs, from the base of their feet, around their toes, all the way up to the top of their legs. Start with the left leg, and then move to the right leg. Slowly visualising each part of the leg.

***Finishing***

And now to finish I would like you to picture in your minds eye where you are seated in this room. Notice and feel the contact your body makes with the chair, and notice how your hands feel by your side. And when you are ready gently open your eyes, and you might like to stretch out.

And that completes today’s Focused Imagery technique.

Thank you for listening and taking part.

# Supplementary Materials 5 – Script for Procedure

**General Script**

*As participants enter, the experimenter directs them to choose a token from a bag. Individuals will either have a white token or a black token, and will be asked to sit on the corresponding side of the table.*

**Experimenter** Before we begin the study please read the information sheet in-front of you. Once you have read this, please read and sign the consent forms and we will begin the experiment.

*Once consent is obtained, begin the video recording.*

**Experimenter** Today you have all been placed into two groups. This group represents a country called Nasherland, and this group represents a country called Lindithia. You each have a description of your country in front of you. I will also read this aloud, so we know our countries.

*The experimenter reads out both country descriptions to the group.*

**Experimenter** The aim of today is to produce as much food as you can. You create food by assembling the LEGO bricks just like this. Watch me.

*Experimenter flips the image of ‘food’ over for participants to see. Experimenter makes one piece of ‘food’, piece by piece for everyone to see and holds it up to display – then dismantles it to return the pieces to the box.*

**Experimenter** So you need to make sure the LEGO bricks go in the correct colour order. It doesn’t matter how they are designed or what pieces you use. What matters is at the bottom is blue, then green, then yellow, and on top is red. If the colour order is not like this, they will not count as food and will be discarded. You should create as much food as possible. Ultimately, we are looking at creating as much food as possible so no one will starve. You will have five minutes to do this to the best of your ability. Does everyone understand the instructions?

*The experimenter begins the timer for 5 minutes.*

**Experimenter**  You may begin.

*The 5-minute timer ends.*

**Experimenter** Okay, everyone stop what you’re doing. Thank you for completing the task today. We now have a questionnaire for you to fill out.

*Experimenter gives each participant a copy of the questionnaire sheets (includes demographic questionnaire, compassion scales, group scales and high/low resource scales). Experimenter also places a clear box full of individually wrapped sweets in the middle of the table.*

**Experimenter** Here’s some lollies just to make this more enjoyable. Feel free to have as many as you want. Once you have completed the questionnaires please leave them where they are and then you are free to leave.

*The experimenter waits for participants to finish – handing out debrief sheets as they leave*.

# Supplementary Materials 6 – Country Descriptions

You are a resident of Lindithia!

Lindithia is a country with a population of 36,500,000. It has a moderate climate with a rainy season that lasts between March and May, with mostly dry summers. In Lindithia, it is common to see a large number of avian species migrating in the winter, as well as small mammals residing in dense forests. In Lindithia, the predominant language is Lindithian, with 85% of the population reporting that as their primary spoken language. The national delicacy of Lindithia is unique to the south, where locals enjoy peach filled pastries.

You are a resident of Nasherland!

Nasherland is a country with a population of 38,300,000. It has a moderate climate with a rainy season that lasts between September and November, with mostly dry winters. In Nasherland, it is common to see a large number of reptiles, as well as medium sized mammals that congregate closer to the rivers. In Nasherland, the predominant language is Nashen, with 85% of the population reporting that as their primary spoken language. The national delicacy of Nasherland is unique to the north, where locals enjoy hazelnut and orange biscuits.

# Supplementary Materials 7 – Full Questionnaire

**Demographics Questionnaire**

**
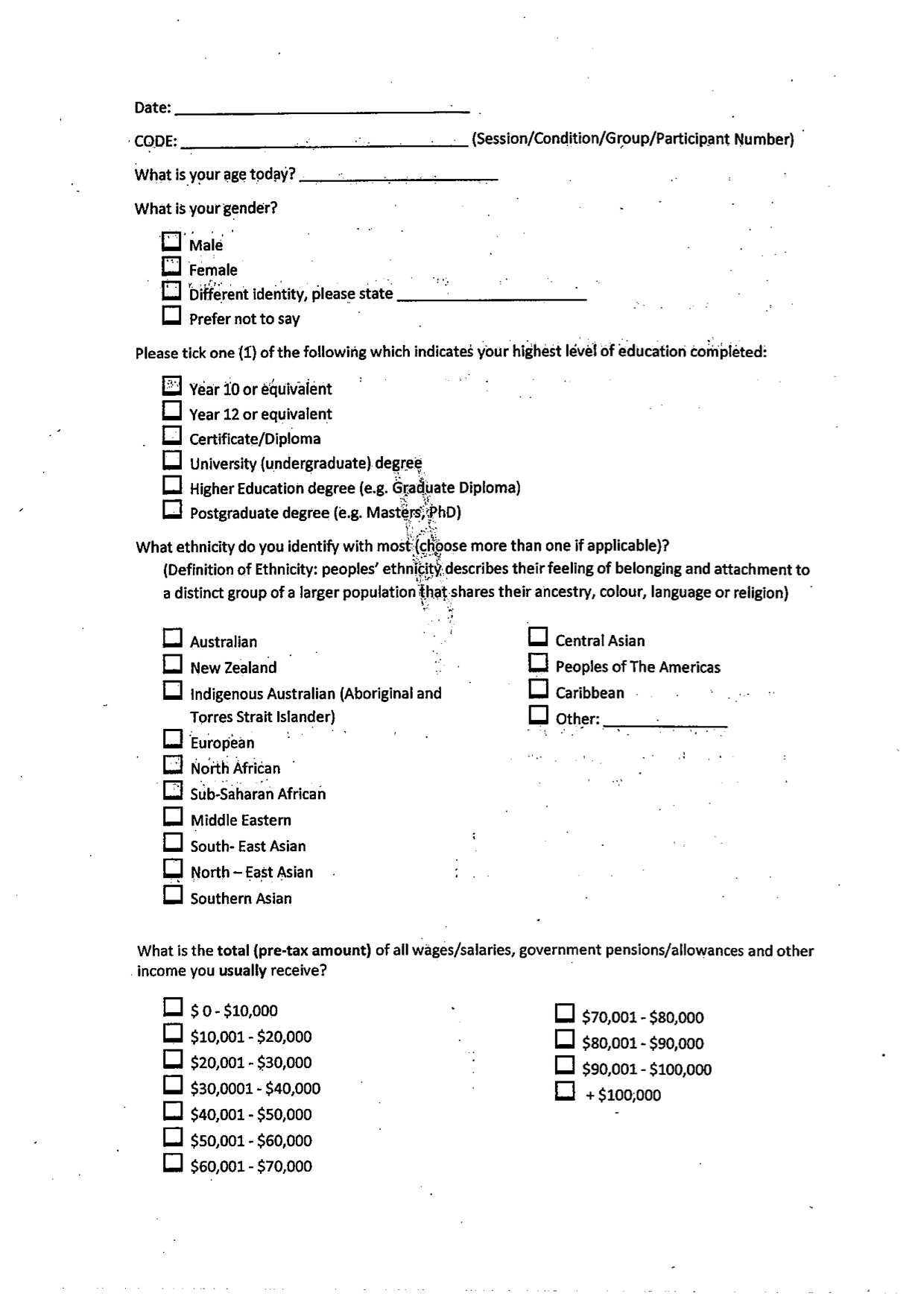
**

**
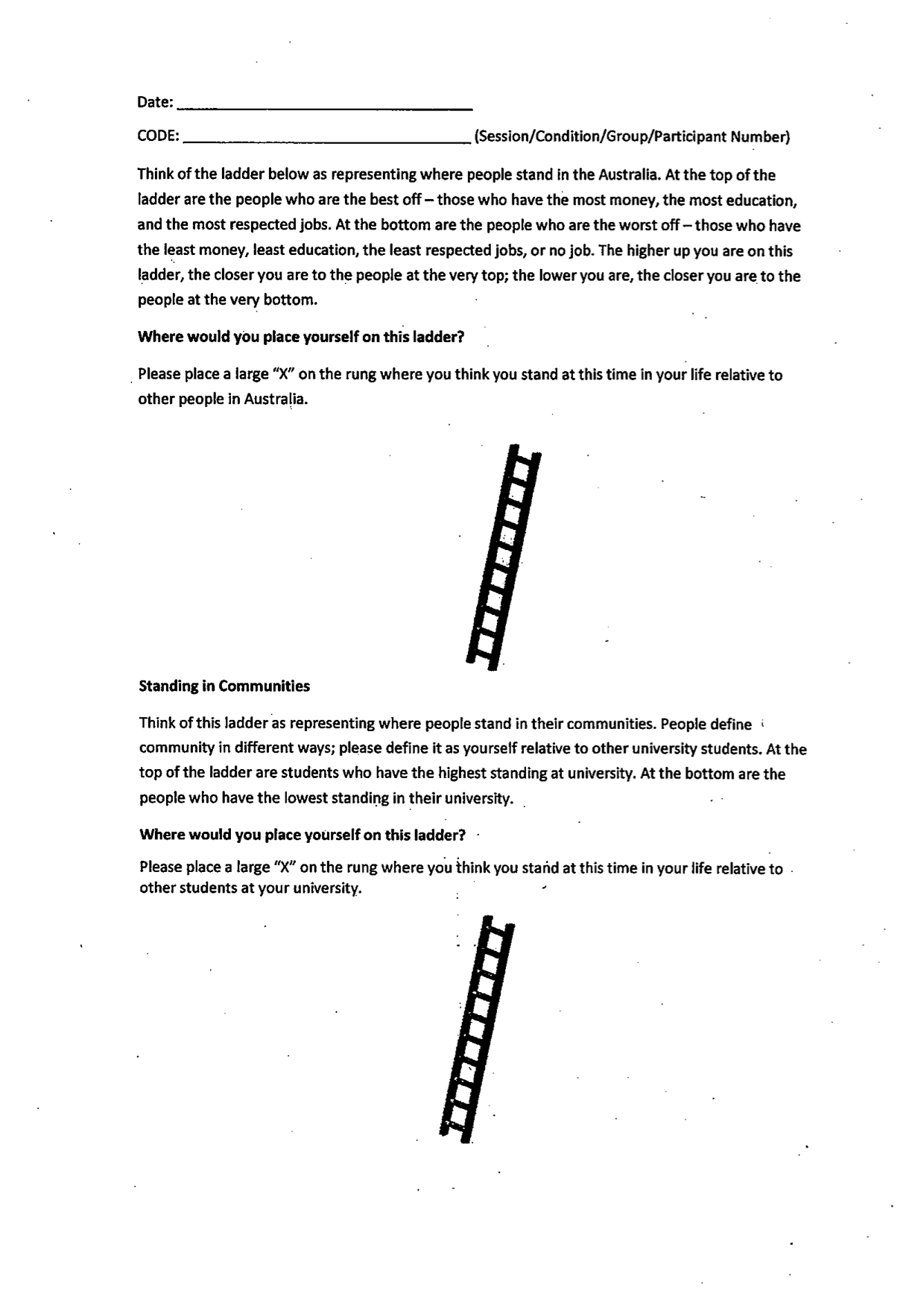
**

**Motivations and Compassion Questionnaire**

Different people have different views of compassion and kindness. While some people believe that it is important to show compassion and kindness in all situations and contexts, others believe we should be more cautious and can worry about showing it too much to ourselves and to others. We are interested in your thoughts and beliefs in regard to kindness and compassion in three areas of your life:

1. Expressing compassion for others
2. Responding to compassion from others

Below are a series of statements that we would like you to think carefully about and then circle the number that best describes how each statement fits you.

**Scale**

***Please use this scale to rate the extent that you agree with each statement***

**Don’t agree at all** 0 1 2 3 4 **Completely Agree**

**Somewhat agree**

**Expressing compassion for others**

**1. Please will take advantage of me if they see me as too compassionate**

0 1 2 3 4

**2. Being compassionate towards people who have done bad things is letting them off the hook**

0 1 2 3 4

**3. There are some people in life who don’t deserve compassion**

0 1 2 3 4

**4. I fear that being too compassionate makes people an easy target**

0 1 2 3 4

**5. People will take advantage of you if you are too forgiving and compassionate**

0 1 2 3 4

**6. I worry that if I am compassionate, vulnerable people can be drawn to me and drain my emotional resources**

0 1 2 3 4

**7. People need to help themselves rather than waiting for others to help them**

0 1 2 3 4

**8. I fear that if I am compassionate, some people will become too dependent upon me**

0 1 2 3 4

**9. Being too compassionate makes people soft and easy to take advantage of**

0 1 2 3 4

**10. For some people, I think discipline and proper punishments are more helpful than being compassionate to them**

0 1 2 3 4

**Responding to compassion from others**

**1. Wanting others to be kind to oneself is a weakness**

0 1 2 3 4

**2. I fear that when I need people to be kind and understanding they won’t be**

0 1 2 3 4

**3. I’m fearful of becoming dependent on the care from others because they might not always be available or willing to give it**

0 1 2 3 4

**4. I often wonder whether displays of warmth and kindness from others are genuine**

0 1 2 3 4

**5. Feelings of kindness from others are somehow frightening**

0 1 2 3 4

**6. When people are kind and compassionate towards me I feel anxious or embarrassed**

0 1 2 3 4

**7. If people are friendly and kind I worry they will find out something bad about me that will change their mind**

0 1 2 3 4

**8. I worry that people are only kind and compassionate if they want something from m**

0 1 2 3 4

**9. When people are kind and compassionate towards me I feel empty and sad**

0 1 2 3 4

**10. If people are kind I feel they are getting too close**

0 1 2 3 4

**11. Even though other people are kind to me, I have rarely felt warmth from my relationships with others**

0 1 2 3 4

**12. I try to keep my distance from others even if I know they are kind**

0 1 2 3 4

**13. If I think someone is being kind and caring towards me, I ‘put up a barrier**

0 1 2 3 4

**Compassion to others**

When things go wrong for other people and they become distressed by setbacks, failures, disappointments or losses, we may cope with their distress in different ways. We are interested in the degree to which people can be **compassionate to others**. We define compassion as “a sensitivity to suffering in self and others with a commitment to try to alleviate and prevent it.” This means there are two aspects to compassion. The first is the ability to be motivated to engage with things/feelings that are difficult as opposed to trying to avoid or suppress them. The second aspect of compassion is the ability to focus on what is helpful. Just like a doctor with his/her patient. The first is to be motivated and able to pay attention to the pain and (learn how to) make sense of it. The second is to be able to take the action that will be helpful. Below is a series of questions that ask you about these two aspects of compassion. Therefore read each statement carefully and think about how it applies to you when people in your life become distressed. Please rate the items using the following rating scale:

**Never Always**

**1 2 3 4 5 6 7 8 9 10**

**Section 1 –These are questions that ask you about how motivated you are, and able to engage with other people’s distress when they are experiencing it. So:**

**When others are distressed or upset by things...**

**1. I am motivated to engage and work with other peoples’ distress when it arises**

Never Always

1 2 3 4 5 6 7 8 9 10

**2. I notice and am sensitive to distress in others when it arises.**

Never Always

1 2 3 4 5 6 7 8 9 10

**3. I avoid thinking about other peoples’ distress, try to distract myself and put it out of my mind.**

Never Always

1 2 3 4 5 6 7 8 9 10

**4. I am emotionally moved by expressions of distress in others**

Never Always

1 2 3 4 5 6 7 8 9 10

**5. I tolerate the various feelings that are part of other people’s distress**

Never Always

1 2 3 4 5 6 7 8 9 10

**6. I reflect on and make sense of other people’s distress**

Never Always

1 2 3 4 5 6 7 8 9 10

**7. I do not tolerate other peoples’ distress**

Never Always

1 2 3 4 5 6 7 8 9 10

**8. I am accepting, non-critical and non-judgemental of others people’s distress**

Never Always

1 2 3 4 5 6 7 8 9 10

**Section 2 –These questions relate to how you actively respond in compassionate ways when other people are distressed. So:**

**When others are distressed or upset by things…**

**1. I direct attention to what is likely to be helpful to others**

Never Always

1 2 3 4 5 6 7 8 9 10

**2. I think about and come up with helpful ways for them to cope with their distress**

Never Always

1 2 3 4 5 6 7 8 9 10

**3. I don’t know how to help other people when they are distressed**

Never Always

1 2 3 4 5 6 7 8 9 10

**4. I take the actions and do the things that will be helpful to others**

Never Always

1 2 3 4 5 6 7 8 9 10

**5. I express feelings of support, helpfulness and encouragement to others**

Never Always

1 2 3 4 5 6 7 8 9 10

Thank you for taking part in our study today. We just have some final questions about your experience of the experiment you just completed.

**To what extent do you agree with the following statements:**

**1. My group was supposed to work independently on the task.**

(Not at all agree) 1 2 3 4 5 6 7 8 9 10 (strongly agree)

**2. My group had enough LEGO bricks to complete the task**

(Not at all agree) 1 2 3 4 5 6 7 8 9 10 (strongly agree)

**3. During the activity, to what extent did you notice the groups were unequal?**

(Not at all aware) 1 2 3 4 5 6 7 8 9 10 (extremely aware)

**4. To what extent did you feel this task was competition between the two countries?**

(Not at all agree) 1 2 3 4 5 6 7 8 9 10 (strongly agree)

**5. To what extent did you feel this task was a cooperative task between the two countries?**

(Not at all agree) 1 2 3 4 5 6 7 8 9 10 (strongly agree)

**6. How well do you remember the aim of the experiment?**

(Don’t remember at all) 1 2 3 4 5 6 7 8 9 10 (Remember very well)

**7. Circle the most accurate response: Was the aim of the experiment to create as much food as you can for everyone?**

- Yes
- No

**8. To what extent did you feel the context was one of “US” (my group) versus “THEM” (the other group)**

(Not at all agree) 1 2 3 4 5 6 7 8 9 10 (strongly agree)

**9. I felt a sense of unity within my group**

(Not at all agree) 1 2 3 4 5 6 7 8 9 10 (strongly agree)

**10. I felt that people in my group seemed to be on the same wavelength**

(Not at all agree) 1 2 3 4 5 6 7 8 9 10 (strongly agree)

**In these sort of experiments it is quite difficult to know what to do and whether to share or not to share. There is no right or wrong answers to these questions, but we wanted to get a sense of what you were feeling during the experiment.**

**Circle the most accurate response:** Did your group share their resources?

- Yes
- No

**If no, to what extent** did you not share because you were worried about the negative reactions of your group members?

(Not at all agree) 1 2 3 4 5 6 7 8 9 10 (strongly agree)

**If no, to what extent** did you not share because you were worried about reducing the wealth/power/status of your group?

(Not at all agree) 1 2 3 4 5 6 7 8 9 10 (strongly agree)

**In these sort of experiments it is quite difficult to know what to do and whether to ask for others to share or not. There is no right or wrong answers to these questions, but we wanted to get a sense of what you were feeling during the experiment.**

**Circle the most accurate response:** Did your group ask for resources to be shared?

- Yes
- No

**If no, to what extent** did you not ask for resources from the other group because you were worried about the negative reactions from the other group (e.g., request would be rejected)?

(Not at all agree) 1 2 3 4 5 6 7 8 9 10 (strongly agree)

**If no, to what extent** did you not ask for resources from the other group because you were worried about the negative reactions from your own group? (e.g., why are you doing that, we can do it ourselves)

(Not at all agree) 1 2 3 4 5 6 7 8 9 10 (strongly agree)

**If no, to what extent** did you not ask for help because you wanted to be able to do it yourself?

(Not at all agree) 1 2 3 4 5 6 7 8 9 10 (strongly agree)

**If you were helped** by the other group did you feel in any way inferior because you couldn’t do it yourself?

(Not at all agree) 1 2 3 4 5 6 7 8 9 10 (strongly agree)

**If you received help, to what extent were you happy to have received help?**

(Not at all agree) 1 2 3 4 5 6 7 8 9 10 (strongly agree)

***To what extent did you listening to the audio track make you feel more compassionate?**

(Not at all agree) 1 2 3 4 5 6 7 8 9 10 (strongly agree)

***How closely were you listening to the audio track?**

(Not at all) 1 2 3 4 5 6 7 8 9 10 (very closely)

*****Questions given for Study 1 only

# Supplementary Materials 8 – Full Results for Each Model

F-statistics were reported in the main manuscript for each Linear Mixed Model (LMM) using the anova() function, and these results can be seen in the R Markdown document on OSF. Below we have additionally provided b estimates and other model information for each LMM, as well as the results from the two-way ANOVAs that were conducted where LMMs yielded singular fit warnings.

**Study 1 – Compassion Meditation**

**Table 7**

Linear Mixed Model Results for Effect of Condition and Resource Group on Noticing Inequality

|  | **Extent to Which Inequality Was Noticed** | | |
| --- | --- | --- | --- |
| *Predictors* | *Estimates* | *CI* | *p* |
| (Intercept) | 6.67 | 6.28, 7.07 | <.001 |
| Condition | 0.30 | -0.10, 0.69 | .137 |
| Resource Group | -0.14 | -0.53, 0.25 | .482 |
| Condition x Resource Group | -0.36 | -0.76, 0.03 | .069 |
| **Random Effects** | | | |
| Residual | 9.84 | | |
| τ_00_ _Group_ | 0.25 | | |
| ICC | .02 | | |
| N _Group_ | 82 | | |
| Observations | 281 | | |
| Marginal R^2^ / Conditional R^2^ | .021 / .045 | | |

**Table 8**

Linear Mixed Model Results for Effect of Condition and Resource Group on Having Enough LEGO Bricks

|  | **Enough LEGO Bricks to Complete Task** | | |
| --- | --- | --- | --- |
| *Predictors* | *Estimates* | *CI* | *p* |
| (Intercept) | 6.16 | 5.88, 6.45 | <.001 |
| Condition | 0.03 | -0.26, 0.31 | .852 |
| Resource Group | 2.54 | 2.26, 2.83 | <.001 |
| Condition x Resource Group | 0.03 | -0.25, 0.31 | .846 |
| **Random Effects** | | | |
| Residual | 5.77 | | |
| τ_00_ _Group_ | 0.00 | | |
| N _Group_ | 82 | | |
| Observations | 282 | | |
| Marginal R^2^ / Conditional R^2^ | .528 / NA | | |

**Table 9**

ANOVA Results for Effect of Condition and Resource Group on Having Enough LEGO Bricks

| Predictor | Sum  of  Squares | *df* | Mean  Square | *F* | *p* | _partial_ η^2^ | _partial_ η^2^  90% CI  [LL, UL] |
| --- | --- | --- | --- | --- | --- | --- | --- |
| (Intercept) | 10619.10 | 1 | 10619.10 | 1840.82 | .000 |  |  |
| Condition | 0.20 | 1 | 0.20 | 0.03 | .852 | .00 | [.00, .01] |
| Resource | 1809.16 | 1 | 1809.16 | 313.62 | .000 | .53 | [.47, .58] |
| Condition x Resource | 0.22 | 1 | 0.22 | 0.04 | .846 | .00 | [.00, .01] |
| Error | 1603.69 | 278 | 5.77 |  |  |  |  |

*Note.* LL and UL represent the lower-limit and upper-limit of the partial η^2^ confidence interval, respectively.

**Table 10**

Estimated Marginal Means for Resource Groups for Feeling as Though Group Had Enough LEGO Bricks

| Resource Group | Mean (*SE*) |
| --- | --- |
| High Resource Group | 8.71(*0.20*) |
| Low Resource Group | 3.62(*0.21*) |

**Table 11**

Linear Mixed Model Results for Effect of Condition and Resource Group on Feelings of Compassion

|  | **Feelings of Compassion** | | |
| --- | --- | --- | --- |
| *Predictors* | *Estimates* | *CI* | *p* |
| (Intercept) | 4.88 | 4.59, 5.16 | <.001 |
| Condition | 0.62 | 0.33, 0.90 | <.001 |
| Resource Group | -0.04 | -0.32, 0.25 | .805 |
| Condition x Resource Group | -0.09 | -0.38, 0.19 | .516 |
| **Random Effects** | | | |
| Residual | 5.60 | | |
| τ_00_ _Group_ | 0.00 | | |
| N _Group_ | 82 | | |
| Observations | 271 | | |
| Marginal R^2^ / Conditional R^2^ | .064 / NA | | |

**Table 12**

ANOVA Results for Effect of Condition and Resource Group on Feelings of Compassion

| Predictor | Sum  of  Squares | *df* | Mean  Square | *F* | *p* | _partial_ η^2^ | _partial_ η^2^  90% CI  [LL, UL] |
| --- | --- | --- | --- | --- | --- | --- | --- |
| (Intercept) | 6383.33 | 1 | 6383.33 | 1139.97 | .000 |  |  |
| Condition | 102.40 | 1 | 102.40 | 18.29 | .000 | .06 | [.02, .12] |
| Resource | 0.34 | 1 | 0.34 | 0.06 | .805 | .00 | [.00, .01] |
| Condition x Resource | 2.37 | 1 | 2.37 | 0.42 | .516 | .00 | [.00, .02] |
| Error | 1495.09 | 267 | 5.60 |  |  |  |  |

*Note.* LL and UL represent the lower-limit and upper-limit of the partial η^2^ confidence interval, respectively.

**Table 13**

Estimated Marginal Means for Conditions in Feelings of Compassion

| Condition | Mean (*SE*) |
| --- | --- |
| Compassion Meditation | 5.49 (*0.21*) |
| Focused Imagery | 4.26 (*0.20*) |

**Table 14**

Generalized Linear Mixed Model Results for Effect of Condition and Resource Group on Sharing (Yes/No)

|  | **Did Sharing Occur** | | |
| --- | --- | --- | --- |
| *Predictors* | *Odds Ratios* | *CI* | *p* |
| (Intercept) | 2364.29 | 134.62, 41523.75 | <.001 |
| Condition | 0.63 | 0.12, 3.37 | .590 |
| Resource Group | 1.89 | 0.35, 10.11 | .456 |
| Condition x Resource Group | 0.49 | 0.09, 2.63 | .407 |
| **Random Effects** | | | |
| Residual | 3.29 | | |
| τ_00_ _Group_ | 62.03 | | |
| ICC | 0.95 | | |
| N _Group_ | 80 | | |
| Observations | 278 | | |
| Marginal R^2^ / Conditional R^2^ | .018 / .951 | | |

**Table 15**

Generalized Linear Mixed Model Results for Effect of Condition and Resource Group on Number of LEGO Bricks Shared

|  | **Number of LEGO Bricks Shared** | | |
| --- | --- | --- | --- |
| *Predictors* | *Incidence Rate Ratios* | *CI* | *p* |
| (Intercept) | 1.95 | 1.43, 2.67 | <.001 |
| Condition | 1.08 | 0.79, 1.47 | .648 |
| Resource Group | 0.82 | 0.60, 1.12 | .204 |
| Condition x Resource Group | 1.24 | 0.90, 1.69 | .184 |
| **Random Effects** | | | |
| Residual | 0.25 | | |
| τ_00_ _Group_ | 1.88 | | |
| ICC | .88 | | |
| N _Group_ | 80 | | |
| Observations | 278 | | |
| Marginal R^2^ / Conditional R^2^ | .042 / .888 | | |

**Table 16**

ANOVA Results for Effect of Condition and Resource Group on Efficiency

| Predictor | Sum  of  Squares | *df* | Mean  Square | *F* | *p* | _partial_ η^2^ | _partial_ η^2^  90% CI  [LL, UL] |
| --- | --- | --- | --- | --- | --- | --- | --- |
| (Intercept) | 3860.37 | 1 | 3860.37 | 720.37 | .000 |  |  |
| Condition | 0.17 | 1 | 0.17 | 0.03 | .857 | .00 | [.00, .03] |
| Resource | 224.14 | 1 | 224.14 | 41.83 | .000 | .35 | [.21, .47] |
| Condition x Resource | 0.45 | 1 | 0.45 | 0.08 | .773 | .00 | [.00, .04] |
| Error | 412.63 | 77 | 5.36 |  |  |  |  |

*Note.* LL and UL represent the lower-limit and upper-limit of the partial η^2^ confidence interval, respectively.

**Table 17**

Estimated Marginal Means for Resource Groups in Efficiency

| Resource Group | Mean (*SE*) |
| --- | --- |
| High Resource | 8.57 (*0.36*) |
| Low Resource | 5.24 (*0.37*) |

**Table 18**

Linear Mixed Model Results for Effect of Condition and Resource Group on Number of Food Pieces Created

|  | **Number of Food Pieces Made** | | |
| --- | --- | --- | --- |
| *Predictors* | *Estimates* | *CI* | *p* |
| (Intercept) | 9.45 | 8.71, 10.19 | <.001 |
| Condition | -0.40 | -1.14, 0.34 | .284 |
| Resource Group | 1.59 | 0.85, 2.33 | <.001 |
| Condition x Resource Group | 0.10 | -0.64, 0.84 | .789 |
| **Random Effects** | | | |
| Residual | 13.15 | | |
| τ_00_ _Group_ | 5.12 | | |
| ICC | .28 | | |
| N _Group_ | 66 | | |
| Observations | 235 | | |
| Marginal R^2^ / Conditional R^2^ | .126 / .371 | | |

**Table 19**

Mean Differences Between Resource Groups in Number of Food Pieces Created

| Resource Group | Mean (*SD*) |
| --- | --- |
| High Resource | 10.79 (*4.93*) |
| Low Resource | 7.83 (*3.46*) |

**Table 20**

Linear Mixed Model Results for Effect of Condition and Resource Group on Fears of Being Compassionate

|  | **Fears of Giving Compassion** | | |
| --- | --- | --- | --- |
| *Predictors* | *Estimates* | *CI* | *p* |
| (Intercept) | 20.12 | 19.16, 21.08 | <.001 |
| Condition | -0.86 | -1.83, 0.10 | .077 |
| Resource Group | -0.16 | -1.13, 0.80 | .734 |
| Condition x Resource Group | 0.00 | -0.96, 0.96 | .996 |
| **Random Effects** | | | |
| Residual | 54.83 | | |
| τ_00_ _Group_ | 2.55 | | |
| ICC | .04 | | |
| N _Group_ | 82 | | |
| Observations | 278 | | |
| Marginal R^2^ / Conditional R^2^ | .013 / .057 | | |

**Table 21**

Linear Mixed Model Results for Effect of Condition and Resource Group on Fears of Receiving Compassion

|  | **Fears of Receiving Compassion** | | |
| --- | --- | --- | --- |
| *Predictors* | *Estimates* | *CI* | *p* |
| (Intercept) | 17.83 | 16.74, 18.93 | <.001 |
| Condition | -0.44 | -1.54, 0.65 | .426 |
| Resource Group | -0.19 | -1.29, 0.90 | .731 |
| Condition x Resource Group | -0.15 | -1.25, 0.94 | .783 |
| **Random Effects** | | | |
| Residual | 83.56 | | |
| τ_00_ _Group_ | 0.00 | | |
| N _Group_ | 82 | | |
| Observations | 273 | | |
| Marginal R^2^ / Conditional R^2^ | .003 / NA | | |

**Table 22**

ANOVA Results for Effect of Condition and Resource Group on Fears of Receiving Compassion

| Predictor | Sum  of  Squares | *df* | Mean  Square | *F* | *p* | _partial_ η^2^ | _partial_ η^2^  90% CI  [LL, UL] |
| --- | --- | --- | --- | --- | --- | --- | --- |
| (Intercept) | 85815.36 | 1 | 85815.36 | 1026.94 | .000 |  |  |
| Condition | 53.04 | 1 | 53.04 | 0.63 | .426 | .00 | [.00, .02] |
| Resource | 9.87 | 1 | 9.87 | 0.12 | .731 | .00 | [.00, .01] |
| Condition x Resource | 6.38 | 1 | 6.38 | 0.08 | .783 | .00 | [.00, .01] |
| Error | 22478.84 | 269 | 83.56 |  |  |  |  |

*Note.* LL and UL represent the lower-limit and upper-limit of the partial η^2^ confidence interval, respectively.

**Table 23**

Linear Mixed Model Results for Effect of Condition and Resource Group on Feelings of Competitiveness

|  | **Context was Competitive** | | |
| --- | --- | --- | --- |
| *Predictors* | *Estimates* | *CI* | *p* |
| (Intercept) | 4.93 | 4.52, 5.35 | <.001 |
| Condition | -0.08 | -0.49, 0.34 | .704 |
| Resource Group | 0.03 | -0.39, 0.44 | .905 |
| Condition x Resource Group | 0.27 | -0.15, 0.68 | .202 |
| **Random Effects** | | | |
| Residual | 8.29 | | |
| τ_00_ _Group_ | 1.06 | | |
| ICC | .11 | | |
| N _Group_ | 82 | | |
| Observations | 282 | | |
| Marginal R^2^ / Conditional R^2^ | .008 / .121 | | |

**Table 24**

Linear Mixed Model Results for Effect of Condition and Resource Group on Feelings of Cooperativeness

|  | **Context was Cooperative** | | |
| --- | --- | --- | --- |
| *Predictors* | *Estimates* | *CI* | *p* |
| (Intercept) | 4.29 | 3.89, 4.69 | <.001 |
| Condition | 0.38 | -0.02, 0.79 | .060 |
| Resource Group | -0.09 | -0.49, 0.31 | .665 |
| Condition x Resource Group | 0.07 | -0.33, 0.47 | .739 |
| **Random Effects** | | | |
| Residual | 6.18 | | |
| τ_00_ _Group_ | 1.43 | | |
| ICC | .19 | | |
| N _Group_ | 82 | | |
| Observations | 281 | | |
| Marginal R^2^ / Conditional R^2^ | .021 / .206 | | |

**Table 25**

Linear Mixed Model Results for Effect of Condition and Resource Group on Feelings the Context was one of Us Versus Them

|  | **Us versus Them** | | |
| --- | --- | --- | --- |
| *Predictors* | *Estimates* | *CI* | *p* |
| (Intercept) | 5.33 | 5.00, 5.65 | <.001 |
| Condition | 0.05 | -0.27, 0.37 | .760 |
| Resource Group | -0.09 | -0.42, 0.23 | .562 |
| Condition x Resource Group | 0.02 | -0.30, 0.35 | .895 |
| **Random Effects** | | | |
| Residual | 7.00 | | |
| τ_00_ _Group_ | 0.11 | | |
| ICC | .02 | | |
| N _Group_ | 82 | | |
| Observations | 282 | | |
| Marginal R^2^ / Conditional R^2^ | .002 / .017 | | |

**Table 26**

Linear Mixed Model Results for Effect of Condition and Resource Group on Feelings of Group Cohesion

|  | **Feeling of Group Cohesion** | | |
| --- | --- | --- | --- |
| *Predictors* | *Estimates* | *CI* | *p* |
| (Intercept) | 6.13 | 5.78, 6.48 | **<.001** |
| Condition | 0.10 | -0.26, 0.45 | .588 |
| Resource Group | -0.84 | -1.19, -0.48 | **<.001** |
| Condition x Resource Group | -0.03 | -0.38, 0.32 | .854 |
| **Random Effects** | | | |
| Residual | 4.19 | | |
| τ_00_ _Group_ | 1.25 | | |
| ICC | .23 | | |
| N _Group_ | 82 | | |
| Observations | 278 | | |
| Marginal R^2^ / Conditional R^2^ | .116 / .318 | | |

**Table 27**

Mean Differences Between Resource Groups in Group Cohesion

| Resource Group | Mean (*SD*) |
| --- | --- |
| High Resource | 5.25 (*2.29*) |
| Low Resource | 6.99 (*2.33*) |

**Study 2 – Altering Inequality**

**Table 28**

Linear Mixed Model Results for Effect of Condition and Resource Group on Noticing Inequality

|  | **Extent to Which Inequality Was Noticed** | | |
| --- | --- | --- | --- |
| *Predictors* | *Estimates* | *CI* | *p* |
| (Intercept) | 7.90 | 7.44, 8.35 | <.001 |
| Condition | 0.34 | -0.11, 0.80 | .139 |
| Resource Group | 0.19 | -0.26, 0.65 | .401 |
| Condition x Resource Group | -0.03 | -0.49, 0.42 | .879 |
| **Random Effects** | | | |
| Residual | 6.99 | | |
| τ_00_ _Group_ | 0.45 | | |
| ICC | .06 | | |
| N _Group_ | 58 | | |
| Observations | 163 | | |
| Marginal R^2^ / Conditional R^2^ | .020 / .080 | | |

**Table 29**

Linear Mixed Model Results for Effect of Condition and Resource Group on Having Enough LEGO Bricks

|  | **Enough LEGO Bricks to Complete Task** | | |
| --- | --- | --- | --- |
| *Predictors* | *Estimates* | *CI* | *p* |
| (Intercept) | 6.11 | 5.56, 6.67 | <.001 |
| Condition | -0.01 | -0.57, 0.54 | .969 |
| Resource Group | 2.29 | 1.74, 2.85 | <.001 |
| Condition x Resource Group | 0.43 | -0.13, 0.98 | .128 |
| **Random Effects** | | | |
| Residual | 5.13 | | |
| τ_00_ _Group_ | 2.50 | | |
| ICC | .33 | | |
| N _Group_ | 58 | | |
| Observations | 163 | | |
| Marginal R^2^ / Conditional R^2^ | .427 / .615 | | |

**Table 30**

Mean Differences Between Resource Groups in Feeling There Was Enough LEGO Bricks to Complete Task

| Resource Group | Mean (*SD*) |
| --- | --- |
| High Resource | 8.34 (*2.53*) |
| Low Resource | 3.84 (*3.06*) |

**Table 31**

Generalized Linear Mixed Model Results for Effect of Condition and Resource Group on Sharing (Yes/No)

|  | **Did Sharing Occur** | | |
| --- | --- | --- | --- |
| *Predictors* | *Odds Ratios* | *CI* | *p* |
| (Intercept) | 49825.06 | 1262.86, 1965807.14 | <.001 |
| Condition | 0.43 | 0.03, 6.42 | .543 |
| Resource Group | 0.84 | 0.06, 12.38 | .896 |
| Condition x Resource Group | 1.23 | 0.08, 18.29 | .879 |
| **Random Effects** | | | |
| Residual | 3.29 | | |
| τ_00_ _Group_ | 705.19 | | |
| ICC | 1.00 | | |
| N _Group_ | 62 | | |
| Observations | 173 | | |
| Marginal R^2^ / Conditional R^2^ | .001 / .995 | | |

**Table 32**

Generalized Linear Mixed Model Results for Effect of Condition and Resource Group on Number of LEGO Bricks Shared

|  | **Number of LEGO Bricks Shared** | | |
| --- | --- | --- | --- |
| *Predictors* | *Incidence Rate Ratios* | *CI* | *p* |
| (Intercept) | 2.89 | 1.82, 4.59 | <.001 |
| Condition | 1.80 | 1.14, 2.86 | .012 |
| Resource Group | 0.50 | 0.32, 0.80 | .004 |
| Condition x Resource Group | 0.81 | 0.51, 1.28 | .365 |
| **Random Effects** | | | |
| Residual | 0.25 | | |
| τ_00_ _Group_ | 3.28 | | |
| ICC | .93 | | |
| N _Group_ | 62 | | |
| Observations | 173 | | |
| Marginal R^2^ / Conditional R^2^ | .197 / .943 | | |

**Table 33**

Mean Differences Between Resource Groups and Conditions in Number of LEGO Bricks Shared

| Resource Group | Mean (*SD*) |
| --- | --- |
| High Resource | 1.71 (*5.52*) |
| Low Resource | 9.22 (*18.18*) |
| Condition |  |
| Extreme Inequality | 8.54 (*17.33*) |
| Standard Inequality | 1.65 (*5.94*) |

**Table 34**

ANOVA Results for Effect of Condition and Resource Group on Efficiency

| Predictor | Sum  of  Squares | *df* | Mean  Square | *F* | *p* | _partial_ η^2^ | _partial_ η^2^  90% CI  [LL, UL] |
| --- | --- | --- | --- | --- | --- | --- | --- |
| (Intercept) | 2853.57 | 1 | 2853.57 | 691.53 | .000 |  |  |
| Condition | 7.14 | 1 | 7.14 | 1.73 | .193 | .03 | [.00, .13] |
| Resource | 106.03 | 1 | 106.03 | 25.70 | .000 | .31 | [.15, .44] |
| Condition x Resource | 1.41 | 1 | 1.41 | 0.34 | .561 | .01 | [.00, .07] |
| Error | 239.33 | 58 | 4.13 |  |  |  |  |

*Note.* LL and UL represent the lower-limit and upper-limit of the partial η^2^ confidence interval, respectively.

**Table 35**

Estimated Marginal Means for Resource Groups in Efficiency

| Resource Group | Mean (*SE*) |
| --- | --- |
| High Resource | 8.10 (*0.37*) |
| Low Resource | 5.48 (*0.37*) |

**Table 36**

Linear Mixed Model Results for Effect of Condition and Resource Group on Number of Food Pieces Created

|  | **Number of Food Pieces Made** | | |
| --- | --- | --- | --- |
| *Predictors* | *Estimates* | *CI* | *p* |
| (Intercept) | 11.26 | 10.28, 12.23 | <.001 |
| Condition | -0.90 | -1.87, 0.07 | .070 |
| Resource Group | 2.47 | 1.50, 3.45 | <.001 |
| Condition x Resource Group | 0.09 | -0.88, 1.07 | .849 |
| **Random Effects** | | | |
| Residual | 18.46 | | |
| τ_00_ _Group_ | 7.73 | | |
| ICC | .30 | | |
| N _Group_ | 62 | | |
| Observations | 173 | | |
| Marginal R^2^ / Conditional R^2^ | .210 / .443 | | |

**Table 37**

Mean Differences Between Resource Groups in Number of Food Pieces Created

| Resource Group | Mean (*SD*) |
| --- | --- |
| High Resource | 13.47 (*4.95*) |
| Low Resource | 8.55 (*5.26*) |

**Table 38**

Linear Mixed Model Results for Effect of Condition and Resource Group on Fears of Being Compassionate

|  | **Fears of Giving Compassion** | | |
| --- | --- | --- | --- |
| *Predictors* | *Estimates* | *CI* | *p* |
| (Intercept) | 19.96 | 18.67, 21.25 | <.001 |
| Condition | -0.04 | -1.33, 1.26 | .957 |
| Resource Group | 1.15 | -0.14, 2.44 | .080 |
| Condition x Resource Group | 0.60 | -0.69, 1.89 | .354 |
| **Random Effects** | | | |
| Residual | 44.74 | | |
| τ_00_ _Group_ | 7.34 | | |
| ICC | .14 | | |
| N _Group_ | 60 | | |
| Observations | 159 | | |
| Marginal R^2^ / Conditional R^2^ | .034 / .170 | | |

**Table 39**

Linear Mixed Model Results for Effect of Condition and Resource Group on Fears of Receiving Compassion

|  | **Fears of Receiving Compassion** | | |
| --- | --- | --- | --- |
| *Predictors* | *Estimates* | *CI* | *p* |
| (Intercept) | 17.04 | 15.48, 18.61 | <.001 |
| Condition | -0.51 | -2.08, 1.06 | .516 |
| Resource Group | 2.10 | 0.54, 3.67 | .009 |
| Condition x Resource Group | 0.56 | -1.00, 2.13 | .475 |
| **Random Effects** | | | |
| Residual | 87.09 | | |
| τ_00_ _Group_ | 3.10 | | |
| ICC | .03 | | |
| N _Group_ | 60 | | |
| Observations | 160 | | |
| Marginal R^2^ / Conditional R^2^ | .055 / .087 | | |

**Table 40**

Mean Differences Between Resource Groups in Fears of Receiving Compassion

| Resource Group | Mean (*SD*) |
| --- | --- |
| High Resource | 19.11 (*10.24*) |
| Low Resource | 14.84 (*8.49*) |

**Table 41**

Linear Mixed Model Results for Effect of Condition and Resource Group on Feelings of Competitiveness

|  | **Context was Competitive** | | |
| --- | --- | --- | --- |
| *Predictors* | *Estimates* | *CI* | *p* |
| (Intercept) | 5.52 | 5.03, 6.01 | <.001 |
| Condition | -0.23 | -0.72, 0.25 | .340 |
| Resource Group | -0.31 | -0.80, 0.17 | .202 |
| Condition x Resource Group | -0.22 | -0.71, 0.27 | .369 |
| **Random Effects** | | | |
| Residual | 8.46 | | |
| τ_00_ _Group_ | 0.33 | | |
| ICC | .04 | | |
| N _Group_ | 58 | | |
| Observations | 162 | | |
| Marginal R^2^ / Conditional R^2^ | .025 / .061 | | |

**Table 42**

Linear Mixed Model Results for Effect of Condition and Resource Group on Feelings of Cooperativeness

|  | **Context was Cooperative** | | |
| --- | --- | --- | --- |
| *Predictors* | *Estimates* | *CI* | *p* |
| (Intercept) | 4.06 | 3.50, 4.62 | <.001 |
| Condition | 0.39 | -0.17, 0.94 | .170 |
| Resource Group | 0.10 | -0.46, 0.66 | .722 |
| Condition x Resource Group | -0.16 | -0.72, 0.40 | .563 |
| **Random Effects** | | | |
| Residual | 6.58 | | |
| τ_00_ _Group_ | 2.00 | | |
| ICC | .23 | | |
| N _Group_ | 58 | | |
| Observations | 163 | | |
| Marginal R^2^ / Conditional R^2^ | .020 / .248 | | |

**Table 43**

Linear Mixed Model Results for Effect of Condition and Resource Group on Feelings the Context was one of Us Versus Them

|  | **Us versus Them** | | |
| --- | --- | --- | --- |
| *Predictors* | *Estimates* | *CI* | *p* |
| (Intercept) | 5.76 | 5.33, 6.19 | <.001 |
| Condition | -0.11 | -0.54, 0.32 | .616 |
| Resource Group | -0.26 | -0.68, 0.17 | .237 |
| Condition x Resource Group | 0.17 | -0.26, 0.59 | .444 |
| **Random Effects** | | | |
| Residual | 7.51 | | |
| τ_00_ _Group_ | 0.00 | | |
| N _Group_ | 58 | | |
| Observations | 163 | | |
| Marginal R^2^ / Conditional R^2^ | .012 / NA | | |

**Table 44**

ANOVA Results for Effect of Condition and Resource Group on Feelings the Context was one of Us Versus Them

| Predictor | Sum  of  Squares | *df* | Mean  Square | *F* | *p* | _partial_ η^2^ | _partial_ η^2^  90% CI  [LL, UL] |
| --- | --- | --- | --- | --- | --- | --- | --- |
| (Intercept) | 5321.94 | 1 | 5321.94 | 708.43 | .000 |  |  |
| Condition | 1.89 | 1 | 1.89 | 0.25 | .616 | .00 | [.00, .03] |
| Resource | 10.60 | 1 | 10.60 | 1.41 | .237 | .01 | [.00, .05] |
| Condition x Resource | 4.43 | 1 | 4.43 | 0.59 | .444 | .00 | [.00, .03] |
| Error | 1194.45 | 159 | 7.51 |  |  |  |  |

*Note.* LL and UL represent the lower-limit and upper-limit of the partial η^2^ confidence interval, respectively.

**Table 45**

Linear Mixed Model Results for Effect of Condition and Resource Group on Feelings of Group Cohesion

|  | **Feeling of Group Cohesion** | | |
| --- | --- | --- | --- |
| *Predictors* | *Estimates* | *CI* | *p* |
| (Intercept) | 6.59 | 6.09, 7.09 | **<.001** |
| Condition | 0.63 | 0.13, 1.13 | **.014** |
| Resource Group | -0.16 | -0.66, 0.33 | .514 |
| Condition x Resource Group | -0.20 | -0.69, 0.30 | .434 |
| **Random Effects** | | | |
| Residual | 2.86 | | |
| τ_00_ _Group_ | 2.46 | | |
| ICC | .46 | | |
| N _Group_ | 58 | | |
| Observations | 163 | | |
| Marginal R^2^ / Conditional R^2^ | .078 / .505 | | |

**Table 46**

Mean Differences Between Conditions in Feelings of Group Cohesion

| Condition | Mean (*SD*) |
| --- | --- |
| Extreme Inequality | 7.24 (*2.32*) |
| Standard Inequality | 6.04 (*2.22*) |

**Study 3 ­– Superordinate Norm**

**Table 47**

Linear Mixed Model Results for Effect of Condition and Resource Group on Noticing Inequality

|  | **Extent to Which Inequality Was Noticed** | | |
| --- | --- | --- | --- |
| *Predictors* | *Estimates* | *CI* | *p* |
| (Intercept) | 7.54 | 6.05, 9.03 | <.001 |
| Condition | 0.55 | 0.11, 0.99 | .016 |
| Resource Group | -0.18 | -0.58, 0.22 | .374 |
| Group Size | 0.23 | -0.30, 0.77 | .381 |
| Condition x Resource Group | 0.14 | -0.25 – 0.53 | .480 |
| **Random Effects** | | | |
| Residual | 5.57 | | |
| τ_00_ _Group_ | 0.17 | | |
| ICC | .03 | | |
| N _Group_ | 64 | | |
| Observations | 160 | | |
| Marginal R^2^ / Conditional R^2^ | .051 / .080 | | |

**Table 48**

Mean Differences Between Conditions on Noticing Inequality

| Condition | Mean (*SD*) |
| --- | --- |
| Extreme Inequality | 8.64 (*1.92*) |
| Standard Inequality | 7.75 (*2.72*) |

**Table 49**

Linear Mixed Model Results for Effect of Condition and Resource Group on Having Enough LEGO Bricks

|  | **Enough LEGO Bricks to Complete Task** | | |
| --- | --- | --- | --- |
| *Predictors* | *Estimates* | *CI* | *p* |
| (Intercept) | 7.22 | 6.04, 8.39 | <.001 |
| Condition | -0.28 | -0.63, 0.07 | .115 |
| Resource Group | 3.12 | 2.80, 3.44 | <.001 |
| Group Size | -0.43 | -0.85, -0.02 | .042 |
| Condition x Resource Group | -0.13 | -0.44, 0.18 | .418 |
| **Random Effects** | | | |
| Residual | 3.87 | | |
| τ_00_ _Group_ | 0.00 | | |
| N _Group_ | 64 | | |
| Observations | 159 | | |
| Marginal R^2^ / Conditional R^2^ | .731 / NA | | |

**Table 50**

ANOVA Results for Effect of Condition and Resource Group on Having Enough LEGO Bricks

| Predictor | Sum  of  Squares | *df* | Mean  Square | *F* | *p* | _partial_ η^2^ | _partial_ η^2^  90% CI  [LL, UL] |
| --- | --- | --- | --- | --- | --- | --- | --- |
| (Intercept) | 569.19 | 1 | 569.19 | 147.15 | .000 |  |  |
| Condition | 9.71 | 1 | 9.71 | 2.51 | .115 | .02 | [.00, .06] |
| Resource | 1431.80 | 1 | 1431.80 | 370.15 | .000 | .71 | [.64, .75] |
| Group Size | 16.24 | 1 | 16.24 | 4.20 | .042 | .03 | [.00, .08] |
| Condition x Resource | 2.56 | 1 | 2.56 | 0.66 | .418 | .00 | [.00, .04] |
| Error | 595.69 | 154 | 3.87 |  |  |  |  |

*Note.* LL and UL represent the lower-limit and upper-limit of the partial η^2^ confidence interval, respectively.

**Table 51**

Estimated Marginal Means for Resource Groups on Having Enough LEGO Bricks

| Resource Group | Mean (*SE*) |
| --- | --- |
| High Resource Group | 9.14 (*0.24*) |
| Low Resource Group | 2.90 (*0.22*) |

**Table 52**

Generalized Linear Mixed Model Results for Effect of Condition and Resource Group on Sharing (Yes/No)

|  | **Did Sharing Occur** | | |
| --- | --- | --- | --- |
| *Predictors* | *Odds Ratios* | *CI* | *p* |
| (Intercept) | 4.45 | 0.01, 1761.75 | .625 |
| Condition | 0.00 | 0.00, 0.02 | <.001 |
| Resource Group | 0.94 | 0.17, 5.25 | .945 |
| Group Size | 0.75 | 0.08, 7.22 | .805 |
| Condition x Resource Group | 0.97 | 0.20, 4.82 | .972 |
| **Random Effects** | | | |
| Residual | 3.29 | | |
| τ_00_ _Group_ | 106.65 | | |
| ICC | .97 | | |
| N _Group_ | 64 | | |
| Observations | 160 | | |
| Marginal R^2^ / Conditional R^2^ | .319 / .980 | | |

**Table 53**

Number of times Sharing Occurred by Condition

| Condition | Yes (*n*) | No (*n*) |
| --- | --- | --- |
| Superordinate Norm | 46 | 26 |
| Control | 19 | 69 |

**Table 54**

Generalized Linear Mixed Model Results for Effect of Condition and Resource Group on Number of LEGO Bricks Shared

|  | **Number of LEGO Bricks Shared** | | |
| --- | --- | --- | --- |
| *Predictors* | *Incidence Rate Ratios* | *CI* | *p* |
| (Intercept) | 1.23 | 0.20, 7.59 | .827 |
| Condition | 2.88 | 1.66, 5.00 | <.001 |
| Resource Group | 1.26 | 0.76, 2.07 | .375 |
| Group Size | 1.58 | 0.79, 3.17 | .194 |
| Condition x Resource Group | 1.02 | 0.64, 1.64 | .926 |
| **Random Effects** | | | |
| Residual | 0.25 | | |
| τ_00_ _Group_ | 3.53 | | |
| ICC | .93 | | |
| N _Group_ | 64 | | |
| Observations | 160 | | |
| Marginal R^2^ / Conditional R^2^ | .190 / .946 | | |

**Table 55**

Mean Differences Between Resource Groups on Number of LEGO Bricks Shared

| Condition | Mean (*SD*) |
| --- | --- |
| Superordinate Norm | 11.00 (*22.92*) |
| Control | 1.94 (*8.20*) |

**Table 56**

ANOVA Results for Effect of Condition and Resource Group on Efficiency

| Predictor | Sum  of  Squares | *df* | Mean  Square | *F* | *p* | _partial_ η^2^ | _partial_ η^2^  90% CI  [LL, UL] |
| --- | --- | --- | --- | --- | --- | --- | --- |
| (Intercept) | 41.08 | 1 | 41.08 | 17.69 | .000 |  |  |
| Condition | 13.84 | 1 | 13.84 | 5.96 | .018 | .09 | [.01, .22] |
| Resource | 205.46 | 1 | 205.46 | 88.50 | .000 | .60 | [.46, .69] |
| Group Size | 53.94 | 1 | 53.94 | 23.23 | .000 | .28 | [.13, .42] |
| Condition x Resource | 3.16 | 1 | 3.16 | 1.36 | .248 | .02 | [.00, .11] |
| Error | 136.98 | 59 | 2.32 |  |  |  |  |

*Note.* LL and UL represent the lower-limit and upper-limit of the partial η^2^ confidence interval, respectively.

**Table 57**

Estimated Marginal Means for Resource Groups on Efficiency

| Resource Group | Mean (*SE*) |
| --- | --- |
| High Resource | 7.04 (*0.28*) |
| Low Resource | 5.95 (*0.30*) |

**Table 58**

Linear Mixed Model Results for Effect of Condition and Resource Group on Number of Food Pieces Created

|  | **Number of Food Pieces Made** | | |
| --- | --- | --- | --- |
| *Predictors* | *Estimates* | *CI* | *p* |
| (Intercept) | 13.64 | 10.79, 16.49 | <.001 |
| Condition | 1.06 | 0.21, 1.90 | .015 |
| Resource Group | 1.68 | 0.90, 2.45 | <.001 |
| Group Size | -1.00 | -2.06, 0.05 | .063 |
| Condition x Resource Group | -0.16 | -0.90, 0.57 | .661 |
| **Random Effects** | | | |
| Residual | 9.09 | | |
| τ_00_ _Group_ | 4.71 | | |
| ICC | .34 | | |
| N _Group_ | 64 | | |
| Observations | 160 | | |
| Marginal R^2^ / Conditional R^2^ | .310 / .546 | | |

**Table 59**

Mean Differences Between Resource Groups and Conditions on Number of Food Pieces Created

| Resource Group | Mean (*SD*) |
| --- | --- |
| High Resource | 12.72 (*4.05*) |
| Low Resource | 8.83 (*3.98*) |
| Condition |  |
| Superordinate Norm | 12.14 (*3.85*) |
| Control | 9.31 (*4.51*) |

**Table 60**

Linear Mixed Model Results for Effect of Condition and Resource Group on Fears of Being Compassionate

|  | **Fears of Giving Compassion** | | |
| --- | --- | --- | --- |
| *Predictors* | *Estimates* | *CI* | *p* |
| (Intercept) | 23.63 | 18.78, 28.48 | <.001 |
| Condition | -1.36 | -2.81, 0.09 | .066 |
| Resource Group | 0.03 | -1.29, 1.36 | .960 |
| Group Size | -1.14 | -2.91, 0.64 | .203 |
| Condition x Resource Group | -1.33 | -2.61, -0.05 | .041 |
| **Random Effects** | | | |
| Residual | 39.13 | | |
| τ_00_ _Group_ | 9.09 | | |
| ICC | .19 | | |
| N _Group_ | 64 | | |
| Observations | 154 | | |
| Marginal R^2^ / Conditional R^2^ | .066 / .242 | | |

**Table 61**

Mean Differences Between Cells on Fears of Being Compassionate

| Resource Group | Condition | Mean (*SD*) |
| --- | --- | --- |
| High Resource | Superordinate Norm | 18.67 (*7.25*) |
| Low Resource | Control | 20.07 (*5.86*) |
| High Resource | Control | 22.90 (*6.84*) |
| Low Resource | Superordinate Norm | 20.62 (*7.73*) |

**Table 62**

Linear Mixed Model Results for Effect of Condition and Resource Group on Fears of Receiving Compassion

|  | **Fears of Receiving Compassion** | | |
| --- | --- | --- | --- |
| *Predictors* | *Estimates* | *CI* | *p* |
| (Intercept) | 19.48 | 12.60, 26.36 | <.001 |
| Condition | 0.95 | -1.09, 2.99 | .353 |
| Resource Group | 0.32 | -1.55, 2.19 | .734 |
| Group Size | -0.50 | -3.02, 2.03 | .694 |
| Condition x Resource Group | -0.83 | -2.64, 0.97 | .360 |
| **Random Effects** | | | |
| Residual | 82.46 | | |
| τ_00_ _Group_ | 16.41 | | |
| ICC | .17 | | |
| N _Group_ | 64 | | |
| Observations | 156 | | |
| Marginal R^2^ / Conditional R^2^ | .025 / .187 | | |

**Table 63**

Linear Mixed Model Results for Effect of Condition and Resource Group on Feelings of Competitiveness

|  | **Context was Competitive** | | |
| --- | --- | --- | --- |
| *Predictors* | *Estimates* | *CI* | *p* |
| (Intercept) | 5.77 | 4.11, 7.43 | <.001 |
| Condition | -0.34 | -0.83, 0.15 | .177 |
| Resource Group | -0.73 | -1.19, -0.28 | .002 |
| Group Size | -0.19 | -0.78, 0.39 | .515 |
| Condition x Resource Group | 0.42 | -0.02, 0.85 | .061 |
| **Random Effects** | | | |
| Residual | 7.72 | | |
| τ_00_ _Group_ | 0.00 | | |
| N _Group_ | 64 | | |
| Observations | 160 | | |
| Marginal R^2^ / Conditional R^2^ | .096 / NA | | |

**Table 64**

ANOVA Results for Effect of Condition and Resource Group on Feelings of Competitiveness

| Predictor | Sum  of  Squares | *df* | Mean  Square | *F* | *p* | _partial_ η^2^ | _partial_ η^2^  90% CI  [LL, UL] |
| --- | --- | --- | --- | --- | --- | --- | --- |
| (Intercept) | 364.89 | 1 | 364.89 | 47.29 | .000 |  |  |
| Condition | 14.20 | 1 | 14.20 | 1.84 | .177 | .01 | [.00, .05] |
| Resource | 79.47 | 1 | 79.47 | 10.30 | .002 | .06 | [.01, .13] |
| Group Size | 3.29 | 1 | 3.29 | 0.43 | .515 | .00 | [.00, .03] |
| Condition x Resource | 27.41 | 1 | 27.41 | 3.55 | .061 | .02 | [.00, .07] |
| Error | 1195.98 | 155 | 7.72 |  |  |  |  |

*Note.* LL and UL represent the lower-limit and upper-limit of the partial η^2^ confidence interval, respectively.

**Table 65**

Estimated Marginal Means for Resource Groups on Feelings of Competitiveness

| Resource Group | Mean (*SE*) |
| --- | --- |
| High Resource | 4.50 (*0.33*) |
| Low Resource | 5.96 (*0.30*) |

**Table 66**

Linear Mixed Model Results for Effect of Condition and Resource Group on Feelings of Cooperativeness

|  | **Context was Cooperative** | | |
| --- | --- | --- | --- |
| *Predictors* | *Estimates* | *CI* | *p* |
| (Intercept) | 4.74 | 2.76, 6.72 | <.001 |
| Condition | 1.30 | 0.71, 1.89 | <.001 |
| Resource Group | 0.05 | -0.49, 0.58 | .866 |
| Group Size | 0.05 | -0.68, 0.78 | .886 |
| Condition x Resource Group | -0.03 | -0.54, 0.49 | .922 |
| **Random Effects** | | | |
| Residual | 5.30 | | |
| τ_00_ _Group_ | 2.04 | | |
| ICC | .28 | | |
| N _Group_ | 64 | | |
| Observations | 160 | | |
| Marginal R^2^ / Conditional R^2^ | .182 / .409 | | |

**Table 67**

Mean Differences Between Conditions on Feelings of Cooperativeness

| Condition | Mean (*SD*) |
| --- | --- |
| Superordinate Norm | 6.21 (*2.64*) |
| Control | 3.56 (*2.68*) |

**Table 68**

Linear Mixed Model Results for Effect of Condition and Resource Group on Feelings the Context was one of Us Versus Them

|  | **Us versus Them** | | |
| --- | --- | --- | --- |
| *Predictors* | *Estimates* | *CI* | *p* |
| (Intercept) | 5.63 | 3.95, 7.32 | <.001 |
| Condition | -0.57 | -1.07, -0.07 | .025 |
| Resource Group | -0.44 | -0.90, 0.02 | .060 |
| Group Size | -0.06 | -0.67, 0.55 | .837 |
| Condition x Resource Group | 0.36 | -0.08, 0.80 | .110 |
| **Random Effects** | | | |
| Residual | 6.55 | | |
| τ_00_ _Group_ | 0.44 | | |
| ICC | .06 | | |
| N _Group_ | 64 | | |
| Observations | 160 | | |
| Marginal R^2^ / Conditional R^2^ | .088 / .145 | | |

**Table 69**

Mean Differences Between Conditions on Feelings the Context was one of Us Versus Them

| Condition | Mean (*SD*) |
| --- | --- |
| Superordinate Norm | 4.93 (*2.49*) |
| Control | 6.06 (*2.83*) |

**Table 70**

Linear Mixed Model Results for Effect of Condition and Resource Group on Feelings of Group Cohesion

|  | **Feeling of Group Cohesion** | | |
| --- | --- | --- | --- |
| *Predictors* | *Estimates* | *CI* | *p* |
| (Intercept) | 4.36 | 2.52, 6.20 | **<.001** |
| Condition | 0.01 | -0.50, 0.52 | .957 |
| Resource Group | -0.07 | -0.54, 0.40 | .758 |
| Group Size | 0.73 | 0.03, 1.44 | **.042** |
| Condition x Resource Group | 0.15 | -0.30, 0.61 | .501 |
| **Random Effects** | | | |
| Residual | 2.72 | | |
| τ_00_ _Group_ | 2.00 | | |
| ICC | .42 | | |
| N _Group_ | 62 | | |
| Observations | 151 | | |
| Marginal R^2^ / Conditional R^2^ | .068 / .463 | | |

# Supplementary Materials 9 – Means and Standard Deviations by Condition, Resource Group and Study

**Table 71**

*Means (Standard Deviations) by Condition and Resource Group for Study 1*

|  | **Compassion Meditation** | | **Focussed Imagery** | |
| --- | --- | --- | --- | --- |
| **Variable** | **High resource** | **Low resource** | **High resource** | **Low resource** |
| Awareness of inequality | 6.47*(3.33)* | 7.46*(3.00)* | 6.60*(3.06)* | 6.17*(3.29)* |
| Enough LEGO Bricks | 8.76*(1.97)* | 3.62*(2.66)* | 8.65*(2.26)* | 3.62*(2.72)* |
| Number of pieces shared | 3.36*(16.12)* | 2.97*(12.44)* | 0.84*(5.01)* | 4.63*(14.29)* |
| Food pieces made | 10.61*(3.72)* | 7.23*(3.61)* | 10.95*(5.89)* | 8.34*(3.27)* |
| Food efficiency* | 8.45*(2.98)* | 5.27*(1.90)* | 8.70*(2.23)* | 5.22*(1.99)* |
| Fears of giving compassion | 19.10*(7.37)* | 19.38*(7.72)* | 20.84*(7.66)* | 21.11*(7.51)* |
| Fears of receiving compassion | 17.04*(9.22)* | 17.73*(9.34)* | 18.24*(9.58)* | 18.31*(8.26)* |
| Feelings of Competitiveness | 5.14*(3.17)* | 4.56*(3.17)* | 4.75*(3.04)* | 5.24*(2.81)* |
| Feelings of Cooperativeness | 4.69*(2.73)* | 4.83*(3.20)* | 3.81*(2.61)* | 4.08*(2.47)* |
| “Us” versus “Them” | 5.31*(2.75)* | 5.44*(2.76)* | 5.16*(2.69)* | 5.39*(2.44)* |
| Group cohesion | 5.35*(2.10)* | 7.11*(2.45)* | 5.17*(2.45)* | 6.87*(2.22)* |
| Feelings of compassion | 5.36*(2.35)* | 5.62*(2.34)* | 4.32*(2.55)* | 4.20*(2.17)* |

*Indicates variable assessed on the group-level

**Table 72**

*Means (Standard Deviations) by Condition and Resource Group for Study 2*

|  | **Extreme Inequality** | | **Moderate Inequality** | |
| --- | --- | --- | --- | --- |
| **Variable** | **High resource** | **Low resource** | **High resource** | **Low resource** |
| Awareness of inequality | 8.42*(2.58)* | 8.09*(2.99)* | 7.79*(2.57)* | 7.32*(2.73)* |
| Enough LEGO Bricks | 8.83*(1.83)* | 3.47*(3.00)* | 7.71*(3.12)* | 4.32*(3.10)* |
| Number of pieces shared | 3.25*(7.29)* | 14.44*(22.73)* | 0.00*(0.00)* | 3.46*(8.29)* |
| Food pieces made | 12.69*(5.55)* | 7.70*(4.96)* | 14.35*(4.08)* | 9.49*(5.47)* |
| Food efficiency* | 7.61*(2.43)* | 5.29*(1.52)* | 8.59*(2.16)* | 5.67*(1.90)* |
| Fears of giving compassion | 21.80*(7.41)* | 18.33*(6.78)* | 20.42*(7.50)* | 19.31*(6.99)* |
| Fears of receiving compassion | 19.17*(10.06)* | 13.90*(8.28)* | 19.03*(10.60)* | 16.03*(8.73)* |
| Feelings of Competitiveness | 4.75*(2.99)* | 5.81*(3.16)* | 5.66*(3.01)* | 5.85*(2.58)* |
| Feelings of Cooperativeness | 4.42*(3.19)* | 4.58*(3.11)* | 3.84*(2.79)* | 3.38*(2.27)* |
| “Us” versus “Them” | 5.56*(2.89)* | 5.74*(3.01)* | 5.45*(2.53)* | 6.29*(2.37)* |
| Group cohesion | 6.94*(2.47)* | 7.57*(2.12)* | 6.09*(2.31)* | 5.99*(2.15)* |

*Indicates variable assessed on the group-level

**Table 73**

*Means (Standard Deviations) by Condition and Resource Group for Study 3*

|  | **Pro-Sharing Group Norm** | | **Control** | |
| --- | --- | --- | --- | --- |
| **Variable** | **High resource** | **Low resource** | **High resource** | **Low resource** |
| Awareness of inequality | 8.55*(2.05)* | 8.72*(1.83)* | 7.37*(2.94)* | 8.09*(2.49)* |
| Enough LEGO Bricks | 9.00*(2.14)* | 2.85*(2.38)* | 9.49*(1.00)* | 2.83(2.1*6)* |
| Number of pieces shared | 12.32*(31.55)* | 10.00*(13.56)* | 2.95*(11.52)* | 1.06*(3.15)* |
| Food pieces made | 14.26*(3.81)* | 10.54*(3.06)* | 11.56*(3.87)* | 7.34*(4.11)* |
| Food efficiency* | 7.81*(2.19)* | 5.23*(1.44)* | 8.40*(1.98)* | 4.67*(1.38)* |
| Fears of giving compassion | 18.67*(7.25)* | 20.62*(7.73)* | 22.90*(6.84)* | 20.07*(5.86)* |
| Fears of receiving compassion | 18.88*(10.32)* | 19.63*(10.89)* | 18.21*(10.24)* | 15.96*(8.15)* |
| Feelings of Competitiveness | 4.70*(2.54)* | 5.26*(2.48)* | 4.39*(3.11)* | 6.62*(2.85)* |
| Feelings of Cooperativeness | 6.24*(2.77)* | 6.18*(2.56)* | 3.63*(2.77)* | 3.49*(2.63)* |
| “Us” versus “Them” | 4.85*(2.73)* | 5.00*(2.31)* | 5.20*(2.82)* | 6.81*(2.64)* |
| Group cohesion | 6.03*(2.20)* | 6.18*(2.30)* | 6.11*(1.92)* | 6.89*(2.30)* |

*Indicates variable assessed on the group-level

# Supplementary Materials 10 – Number of Times Each Sharing Behaviour Occurred

**Study 1**

Table 74

*Number of Times Each Category of Behavior Occurred*

| Category of behaviour | Number of occurrences |
| --- | --- |
| High Resource Group shares spontaneously | 6 |
| High Resource Group discuss sharing | 3 |
| Low Resource Group request help 🡪 immediate help given | 19 |
| Low Resource Group request help 🡪 vague response (no clear giving) | 3 |
| Low Resource Group request help 🡪 none given | 4 |
| Low Resource Group takes without requesting | 6 |
| No Request or No Offer | 186 |
| High Resource asks Low Resource if they would like LEGO bricks | 4 |
| Both groups pool resources and work together | 9 |

**Study 2**

Table 75

*Number of Times Each Category of Behavior Occurred*

| Category of behaviour | Number of occurrences |
| --- | --- |
| High Resource Group shares spontaneously | 6 |
| High Resource Group discuss sharing | 6 |
| Low Resource Group request help 🡪 immediate help given | 11 |
| Low Resource Group request help 🡪 vague response (no clear giving) | 6 |
| Low Resource Group request help 🡪 none given | 0 |
| Low Resource Group takes without requesting | 16 |
| No Request or No Offer | 114 |
| High Resource asks Low Resource if they would like LEGO bricks | 1 |
| Both groups pool resources and work together | 1 |

**Study 3**

Table 76

*Number of Times Each Category of Behavior Occurred*

| Category of behaviour | Number of occurrences |
| --- | --- |
| High Resource Group shares spontaneously | 4 |
| High Resource Group discuss sharing | 16 |
| Low Resource Group request help 🡪 immediate help given | 15 |
| Low Resource Group request help 🡪 vague response (no clear giving) | 3 |
| Low Resource Group request help 🡪 none given | 4 |
| Low Resource Group takes without requesting | 19 |
| No Request or No Offer | 93 |
| High Resource asks Low Resource if they would like LEGO bricks | 13 |
| Both groups pool resources and work together | 6 |

# Supplementary Materials 11 – Study 2 Country Descriptions for Extreme Inequality Condition

You are a resident of Lindithia!

Lindithia is a country with a population of 36,500,000, and most residents are very poor. It has a moderate climate with a rainy season that lasts between March and May, with mostly dry summers. In Lindithia, it is common to see a large number of avian species migrating in the winter, as well as small mammals residing in dense forests. In Lindithia, the predominant language is Lindithian, with 85% of the population reporting that as their primary spoken language. The national delicacy of Lindithia is unique to the south, where locals enjoy peach filled pastries.

You are a resident of Nasherland!

Nasherland is a country with a population of 38,300,000, and most residents are very rich. It has a moderate climate with a rainy season that lasts between September and November, with mostly dry winters. In Nasherland, it is common to see a large number of reptiles, as well as medium sized mammals that congregate closer to the rivers. In Nasherland, the predominant language is Nashen, with 85% of the population reporting that as their primary spoken language. The national delicacy of Nasherland is unique to the north, where locals enjoy hazelnut and orange biscuits.

# Supplementary Materials References

Piff, P. K., Stancato, D. M., Cot̂eb́, S., Mendoza-Denton, R., & Keltner, D. (2012). Higher social class predicts increased unethical behavior. *Proceedings of the National Academy of Sciences of the United States of America*, *109*(11), 4086–4091. https://doi.org/10.1073/pnas.1118373109
